# Elevated CO_2_ reinforces PT11-dependent symbiotic phosphate uptake to reprogram root nutrient acquisition in rice

**DOI:** 10.1073/pnas.2606406123

**Published:** 2026-09-01

**Authors:** Chai Hao Chiu, Mette Grønlund, Thomas Christian de Bang, Stephanie Watts-Williams, Fang Song, Krystyna Anna Kelly, Iver Jakobsen, Uta Paszkowski

**Affiliations:** ^a^ Crop Science Centre, Department of Plant Sciences, University of Cambridge, Cambridge CB3 0LE, United Kingdom; ^b^ Department of Plant and Environmental Sciences, University of Copenhagen, Frederiksberg C DK-1871, Denmark; ^c^ Centre for Crop Systems Analysis, Department of Plant Sciences, Wageningen University & Research, Wageningen 6708 PE, The Netherlands; ^d^ The School of Agriculture, Food and Wine, College of Science, Adelaide University, Urrbrae, SA 5064, Australia; ^e^Department of Plant Sciences, Cambridge CB2 3EA, United Kingdom

**Keywords:** arbuscular mycorrhizal symbiosis, phosphate uptake, elevated CO_2_, transcriptomics, root system

## Abstract

Plants acquire inorganic phosphate (Pi) either directly through their roots or through symbiosis with arbuscular mycorrhizal (AM) fungi, and the direct uptake pathway is downregulated when the symbiosis establishes. As atmospheric CO_2_ concentrations rise, it is critical to understand how increased carbon availability alters plant nutrient acquisition strategies, with implications for crop productivity and carbon sequestration alike. Here, we investigated the interaction between elevated CO_2_, soil Pi availability, and AM symbiosis in the major cereal crop rice. Elevated CO_2_ enhanced mycorrhizal colonization, phosphorus uptake, and crop biomass. We employed the rice *pt11* mutant, which is defective in symbiotic Pi transport, in combination with split-root and radiotracer approaches to demonstrate that suppression of the direct Pi uptake pathways occurs locally in colonized roots, requiring functional symbiotic Pi transport. Likewise, changes in root architecture—notably reduced fine lateral root development—occur with local regulation dependent on processes downstream of PT11. Transcriptomic analyses identify symbiotic Pi transport as a regulatory checkpoint of the suppression of direct nutrient uptake and the progression of the mycorrhizal transcriptional program. Together, these findings reveal that in rice, rising CO_2_ enhances AM symbiosis not necessarily by activating canonical symbiosis signaling pathways, but rather by reinforcing symbiotic Pi uptake through enhanced carbon availability, thereby reshaping root nutrient acquisition. This work provides a mechanistic framework for integrating plant–microbe interactions into strategies aimed at sustaining crop productivity and managing carbon in a high-CO_2_ world.

## Significance

Rising atmospheric CO_2_ is expected to increase crop productivity, yet how it influences nutrient acquisition strategies and plant–microbe interactions remains poorly understood. Using rice as a model cereal, we show that elevated CO_2_ enhances arbuscular mycorrhizal colonization and inorganic phosphate (Pi) acquisition primarily by increasing carbon availability and sustaining functional symbiotic Pi exchange, rather than by activating canonical symbiosis signaling pathways. Symbiotic Pi transport through the mycorrhizal transporter PT11 acts locally to suppress direct Pi uptake, remodel root architecture, and drive progression of symbiotic transcriptional programs. These findings reveal how elevated CO_2_ fundamentally rewires Pi uptake strategies in a cereal and highlight symbiotic nutrient exchange as a key determinant of plant responses to future atmospheric conditions.

Atmospheric carbon dioxide (CO_2_) concentrations are rising rapidly and are projected to exceed 700 ppm within this century (1). Elevated CO_2_ enhances photosynthetic carbon fixation and biomass accumulation in C_3_ crops (2–5), including rice (2, 6, 7), yet these gains are frequently constrained by nutrient availability (7–9). Among limiting nutrients, nitrogen (N) and especially phosphorus (P) impose strong constraints on productivity (10). Because Pi acquisition relies on diffusion-limited processes, plants must coordinate root system architecture, specialized transporters, and symbiotic interactions to meet demand (reviewed in refs. 11 and 12). Despite extensive physiological research (2, 6, 7, 13–15), it remains poorly understood how elevated CO_2_ signals are perceived and translated into root nutrient acquisition strategies (16).

In most terrestrial ecosystems, plants acquire phosphorus through two partially redundant pathways: direct, asymbiotic uptake by roots and indirect uptake mediated by arbuscular mycorrhizal (AM) fungi (reviewed in ref. 12). AM symbiosis represents a major biological pathway for Pi uptake in most land plants, including cereals, by extending the soil exploration capacity of roots. However, symbiosis imposes a substantial carbon cost on the host (17–20), and the balance between direct and symbiotic uptake pathways is tightly regulated by soil Pi availability and host signaling mechanisms (reviewed in ref. 21). Although elevated CO_2_ is typically thought to increase carbon availability to both roots and fungal symbionts, its exact outcome, impact on AM symbiosis, Pi uptake pathway choice, and root development remains unclear. Recent work in *Arabidopsis* has shown that elevated CO_2_ modifies nutrient signaling pathways in roots (16), but also highlights a major knowledge gap: How CO_2_ signals are integrated at the root transcriptional level with biological nutrient acquisition strategies such as symbiosis.

Available studies of elevated CO_2_ report highly variable and context-dependent effects on mycorrhizal colonization (8, 22) with elevated CO_2_ stimulating AM colonization in some (8), and conferring inconsistent benefits in others (23–26). Moreover, most previous work focused on whole-plant outcomes, leaving unresolved how elevated CO_2_ remodels root system architecture, carbon allocation, the relative engagement of direct vs. symbiotic Pi uptake, and plant signaling and transcriptional pathways. Whether CO_2_ enrichment overrides host control of symbiosis, enhances mycorrhizal benefit, or instead promotes direct nutrient foraging thus remains an unresolved question.

Rice offers a powerful system to address these questions because AM fungi colonize only a subset of its root types (27, 28) and symbiotic Pi uptake is mediated by genetically defined transporters (29, 30). In rice, symbiotic Pi uptake is mediated primarily by the mycorrhiza-specific Pi transporter PT11, which localizes to the periarbuscular membrane and functions nonredundantly with the related transporter PT13. Disruption of PT11 and its orthologous genes in other species (e.g., *Medicago PT4*) arrests arbuscule development and severely impairs symbiotic Pi transfer (29, 31, 32). This enables genetic separation of early symbiotic signaling from nutrient uptake and positions PT11 as a potential regulatory checkpoint linking Pi flux to downstream signaling and developmental responses (31, 33). However, how PT11-dependent nutrient acquisition regulates transcriptional and physiological outcomes—under both ambient and elevated CO_2_—remains unexplored (34).

Here, we investigate how elevated atmospheric CO_2_ reshapes Pi acquisition strategies, root system architecture, and AM symbiosis in rice across a soil Pi availability gradient. By combining physiological analyses, split-root systems, dual radioisotope tracing, and transcriptome profiling in wild-type and *pt11* mutant roots, we dissect how CO_2_ availability modulates local and systemic regulation of direct and symbiotic Pi uptake pathways. Our results reveal that elevated CO_2_ enhances AM symbiosis primarily by reinforcing functional symbiotic Pi acquisition rather than by activating canonical symbiosis signaling pathways and that this import acts as a local checkpoint coordinating root architectural remodeling, suppression of direct nutrient uptake, and transcriptional progression. Together, this work resolves, how elevated CO_2_ impacts root physiology and transcription in both asymbiotic and symbiotic contexts and provides a mechanistic framework for understanding how rising atmospheric CO_2_ is translated into root-based outcomes in cereal crops through integration of carbon supply and symbiotic nutrient exchange.

## Results

### Elevated Atmospheric CO_2_ Enhances Rice Root Architecture and Mycorrhizal Symbiosis.

**Fig. 1.**
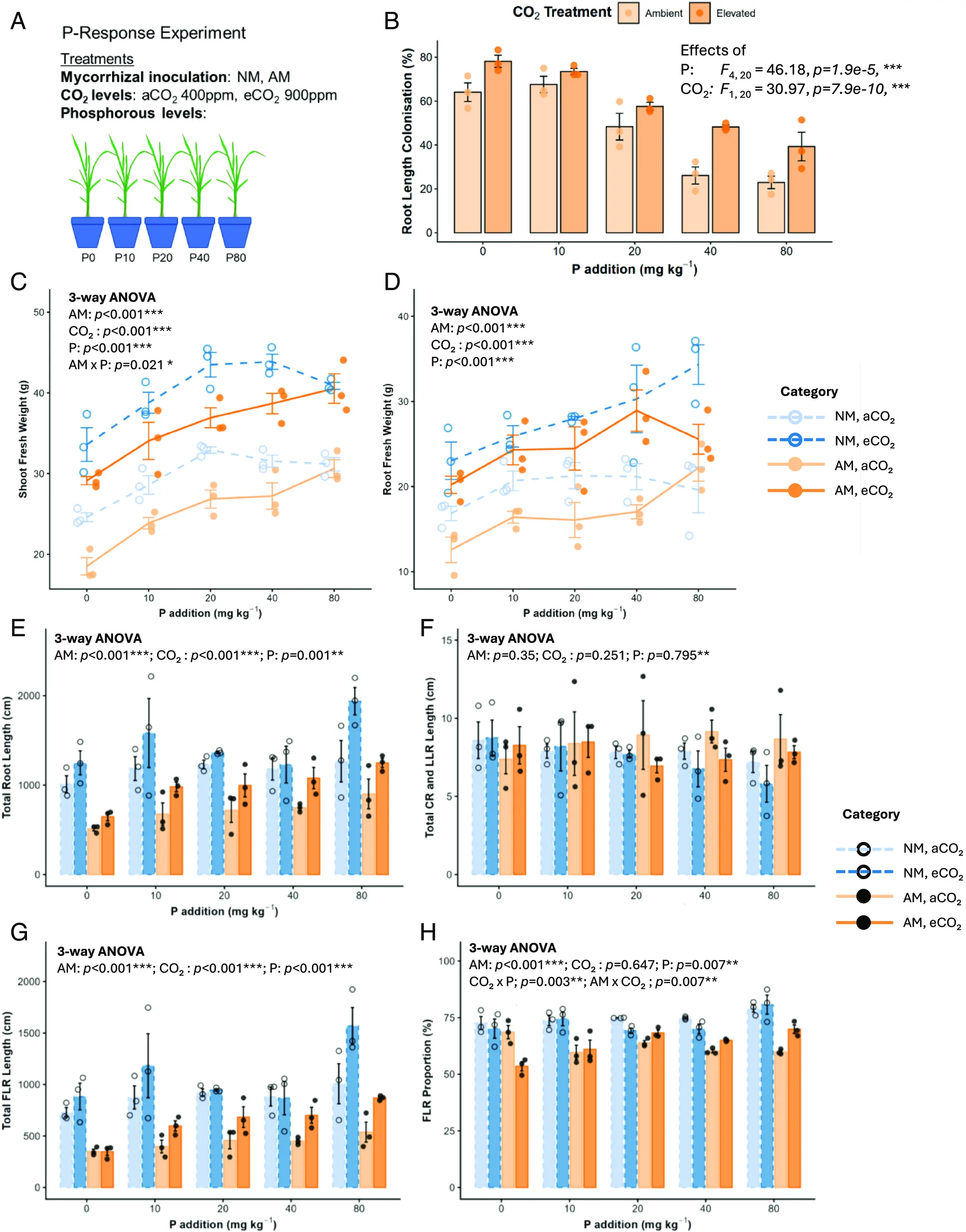
Effects of phosphate availability, arbuscular mycorrhizal symbiosis, and atmospheric CO_2_ on rice growth and root system architecture. (*A*) Schematic of the experimental setup. Rice plants were grown across a gradient of phosphate (P) supply (0 to 80 mg P kg^–1^ soil) under ambient (400 ppm) or elevated (900 ppm) CO_2_, either nonmycorrhizal (NM) or inoculated with arbuscular mycorrhizal (AM) fungi, *Rhizophagus irregularis*. (*B*) Percentage of root length colonized by AM fungi across P levels under ambient (light orange) and elevated CO_2_ (dark orange). Bars represent means ± SEM; points indicate individual biological replicates. Shoot fresh weight (*C*) and root fresh weight (*D*) as a function of P supply. Dashed lines, open circles in blue: NM plants; solid lines, filled circles, and orange shade: AM plants; color intensity representing CO_2_ treatment, with elevated CO_2_ being darker. (*E*) Total root length, (*F*) Combined crown root (CR) and large lateral root (LLR) lengths. (*G*) Total fine lateral root (FLR) length, (*H*) Proportion of total root length contributed by fine lateral roots. Bars represent means ± SEM with individual plants overlaid. Color scheme consistent with above. Text *Insets* provide three-way ANOVA *P*-values for AM inoculation (AM), CO_2_, and P addition (P).

We first examined how the interaction between soil phosphorus (P) availability, AM fungi, and atmospheric CO_2_ influences rice growth and nutrient acquisition. Wild-type rice plants were grown under a gradient of five different levels of P supply (P0 to P80, corresponding to mg of P/kg of soil), either without or with AM fungi (NM and AM) and under ambient (aCO_2,_ 400 ppm) or elevated CO_2_ (eCO_2,_ 900 ppm) conditions (Fig. 1*A*). AM colonization of roots declined progressively with increasing phosphate supply (*P* < 0.001; Fig. 1*B*), consistent with P-mediated suppression of AM symbiosis. Across all P levels except P10, elevated CO_2_ significantly increased level of colonization (*P* < 0.001). This enhancement was more pronounced at higher P levels (P40 and P80), consistent with eCO_2_ partially alleviating P-dependent suppression of symbiosis (Fig. 1*B*). Elevated CO_2_ did not prevent the suppression of symbiosis, indicating that increased carbon availability increased fungal root colonization without overriding host regulatory control. Compared with previous analyses using the same setup in pea, *Medicago truncatula, Zea mays,* and *Brachypodium distachyon* (23–25, 34), rice displayed a stronger CO_2_-dependent stimulation of AM colonization.

P availability strongly constrained plant growth (*P* < 0.001 for shoot fresh weight for instance), with shoot and root biomass increasing up to P20 and generally plateauing thereafter across CO_2_ and mycorrhizal treatments (Fig. 1 *C* and *D* and *SI Appendix*, Fig. S1), the exception being continued root growth up to P80 (Fig. 1*D* and *SI Appendix*, Fig. S1*B*). Elevated CO_2_ significantly increased shoot and root biomass in both NM (nonmycorrhizal) and AM plants at all P levels, confirming CO_2_ as a major growth-limiting factor. Despite this overall stimulation, AM plants consistently accumulated less shoot and root biomass than NM plants under both CO_2_ regimes (−19% at aCO_2_ and −18% at eCO_2_; *P* < 0.001), except at the highest P level where differences were no longer detectable (Fig. 1 *C* and *D* and *SI Appendix*, Fig. S1). This reduction in biomass, independent of CO_2_ and P availability, likely reflects the carbon cost of sustaining AM symbiosis. At high P, where symbiotic nutrient exchange is expected to be low with reduced colonization (Fig. 1*B*), AM fungi may represent a weak carbon sink, explaining the convergence of biomass between NM and AM plants. Together, these results show that the commonly used reference cultivar, Nipponbare, displays a neutral to mildly negative growth response to AM fungi under the conditions used here.

To assess the influence of P availability and AM fungal treatments on root system architecture, we estimated the total root length and its partitioning among root types (Fig. 1 *E*–*H*). The adult rice root system comprises crown roots (CRs) that grow from the shoot and provide anchorage, from which lateral roots (LRs) then emerge. Because AM fungi colonize large LRs but not fine LRs in rice, we dissected root length into CRs and large LRs vs. fine LRs. Total root length increased with both P supply (*P* = 0.001) and CO_2_ availability (*P* < 0.001) but was significantly reduced by AM symbiosis (*P* < 0.001; Fig. 1*E*). Partitioning of root length revealed that this reduction was driven primarily by suppression of fine LRs, both in absolute length (*P* < 0.001, Fig. 1*G*) and proportional contribution (*P* < 0.001; Fig. 1*H*), whereas CRs and large LRs were comparatively less sensitive (*P* = 0.35, Fig. 1*F*). These responses indicate a shift from elongation-based root foraging toward symbiotic nutrient acquisition in colonized roots, with eCO_2_ increasing overall root system size but not overriding AM-induced remodeling of root architecture.

### Elevated CO_2_ Alters Shoot Phosphorous Accumulation and Phosphorous Use Efficiency.

We next quantified shoot P concentration, total shoot P content, and P uptake efficiency (PUE). Both shoot P concentration (*P* < 0.001) and total P content (*P* < 0.001) increased progressively with increasing P supply, indicating improved plant P status across the fertilization gradient (*SI Appendix*, Fig. S2 *A* and *B*). Under aCO_2_, AM plants exhibited higher shoot P concentrations than NM plants at the lowest P levels (P0 and P10), reflecting enhanced P acquisition under nutrient limitation (*P* < 0.01). This difference was not observed at higher P supplies (*SI Appendix*, Fig. S2*A*). In contrast, at P40 and P80, AM plants accumulated significantly less total shoot P than NM plants (*SI Appendix*, Fig. S2*B*), a reduction driven primarily by lower biomass (Fig. 1*C* and *SI Appendix*, Fig. S1*A*) rather than altered tissue P concentration (*SI Appendix*, Fig. S2*A*).

When normalized to root biomass, AM plants accumulated shoot P comparable to NM plants with reduced root investment at low P, resulting in higher root length–specific P uptake (*SI Appendix*, Fig. S2*C*, *P* < 0.001). PUE increased under eCO_2_ (*P* < 0.01) but was reduced by AM colonization at a given CO_2_ level (*P* < 0.01) at P0-20, reflecting CO_2_-driven biomass gains and the carbon cost of symbiosis (*SI Appendix*, Fig. S2*D*). Indeed, eCO_2_ significantly increased total shoot P content (*SI Appendix*, Fig. S2*B*) owing to the substantial increase in plant biomass. However, shoot P concentration generally decreased under eCO_2_ across P treatments in both NM and AM plants, except at the highest P supply, consistent with dilution effects associated with enhanced growth. These data indicate that AM symbiosis improves P acquisition efficiency under limitation but has a negative impact when P supply is higher; whereas eCO_2_ primarily increases total P accumulation through enhanced growth.

### Root System Remodeling Involves Both Local and Systemic Signaling.

**Fig. 2.**
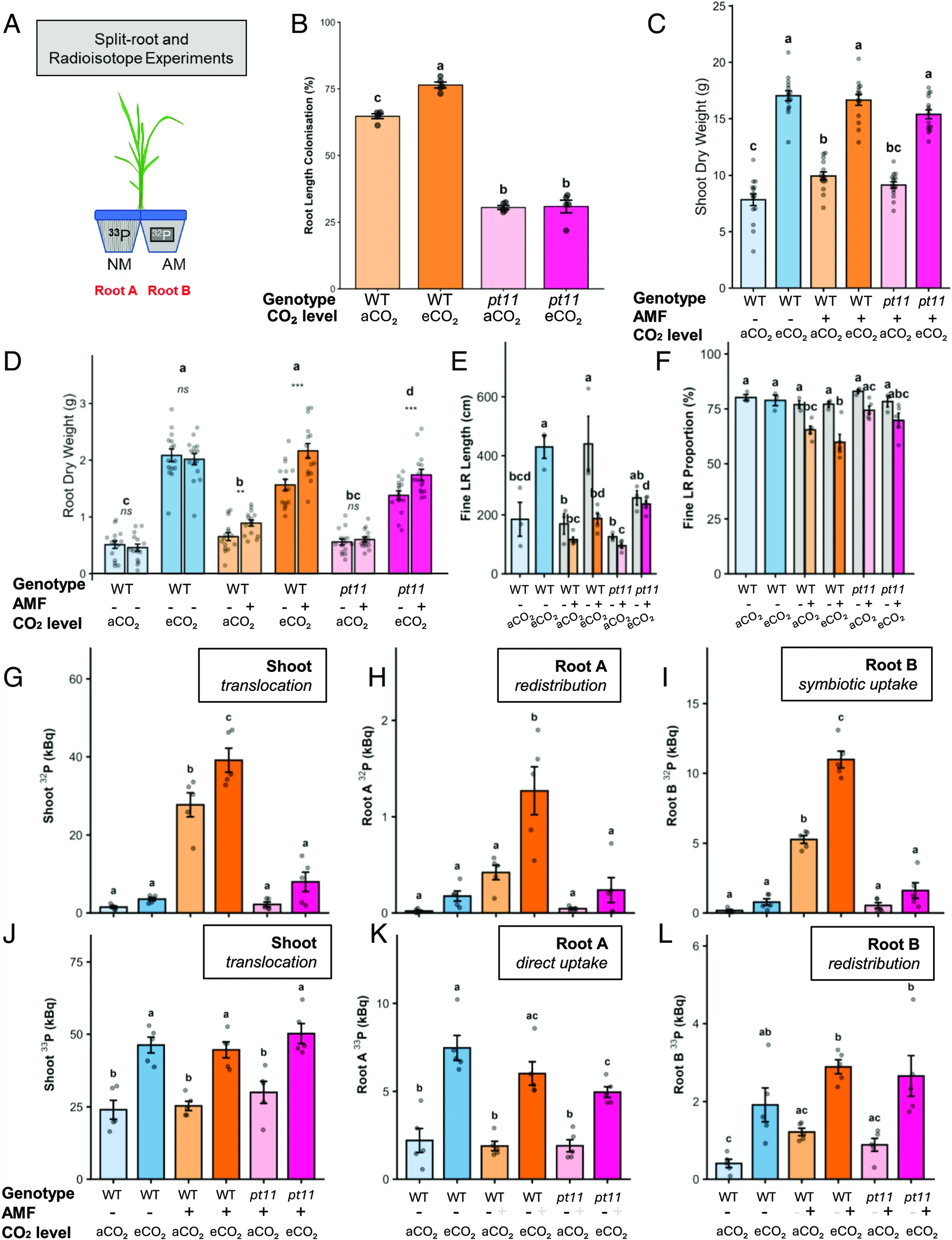
Elevated CO_2_ and PT11 regulate root growth, phosphate allocation, and direct vs. symbiotic phosphate uptake in a split-root system. Wild-type (WT) and *pt11-1* mutant plants were grown in a split-root system in which one root compartment (Root A) remained nonmycorrhizal (NM) and the other compartment (Root B) was either NM or colonized by AM fungi (AM). Plants were cultivated under ambient or elevated CO_2_ at P20 phosphate supply. (*A*) Schematic of the split-root system and dual radioisotope labeling strategy. ^33^P was supplied to the NM compartment (Root A) to trace direct phosphate uptake, while ^32^P was supplied to the AM compartment (Root B) via a hyphal-only chamber to trace symbiotic uptake. (*B*) Percentage of root length colonized by AM fungi in mycorrhizal root compartments across genotypes and CO_2_ treatments. Different letters denote significant differences (*P* < 0.05, Tukey’s HSD) (*C*) Shoot dry weight of WT and *pt11* plants grown in the split-root experiments under ambient or elevated CO_2_, with or without AM colonization. (*D*) Dry weight of NM roots (Root A), and AM roots (Root B) shown as paired bars. (*E*) Fine lateral root (FLR) length, (*F*) FLR proportion. Paired bars show roots in compartments A and B in a split-root setup. Root architectural traits were quantified separately for each root compartment. (*G*–*I*) Distribution of ^32^P-derived phosphate (symbiotic uptake) in shoots (*G*), Root A (*H*), and Root B (*I*, where it was supplied to AM fungal hyphae). (*J*–*L*) Distribution of ^33^P -derived phosphate (direct uptake) in shoots (*J*), Root A (*K*, where it was supplied), and Root B (*L*). Bars represent means ± SEM; individual data points represent biological replicates. Color scheme consistent with above, blue: NM plants, orange: mycorrhizal plants, gray: NM half of an AM colonized plant, color intensity: CO_2_ treatment, with elevated CO_2_ being darker. Note: bars in (*D*) are all paired within-plant measurements; gray shading in (*E* and *F*) distinguishes paired bars (NM/AM) split-root measurements from unpaired (NM/NM) mock controls.

Root responses to eCO_2_ and AM symbiosis reflect coordinated regulation of carbon supply and P demand between shoots and roots. To distinguish local from systemic control of root architecture and Pi uptake pathways, we used a split-root system and exploited the requirement of the symbiotic Pi transporter OsPT11 for arbuscule function by using *ospt11-1* mutant (characterized in ref. 29; hereafter ***pt11***). One root compartment was nonmycorrhizal (Root A) while the other was colonized by AM fungi (Root B), as represented in Fig. 2*A*. Plants were grown at intermediate P supply (P20) under ambient or elevated CO_2_.

Increased AM colonization under eCO_2_ was recapitulated in wild-type but not *pt11* (Fig. 2*B*), suggesting that sustained symbiotic nutrient exchange might underpin the enhanced colonization observed under eCO_2_. Elevated CO_2_ increased shoot biomass in both genotypes (*P* < 0.001; Fig. 2*C*), and also root biomass, particularly in NM wild-type plants (fourfold increase), in both compartments, root A and root B (Fig. 2*D*).

The split-root system further allowed the analysis of resource allocation to root growth when one compartment (Root A) is nonmycorrhizal while the other compartment (Root B) is mycorrhizal. Under aCO_2_, AM colonization tended to increase root biomass specifically of the AM compartment in wild-type plants but not in *pt11* (Fig. 2*D*), indicating a local *PT11*-dependent response. Under eCO_2_, however, total root growth increased and preferential allocation to the colonized compartment was restored in *pt11* (Fig. 2*D*, *P* < 0.001), indicating that symbiotic Pi transfer is partially required for this redistribution of root growth.

Root architectural analysis identified fine LRs as the most responsive root architecture change, as compared to CRs and large LRs (Fig. 2 *E* and *F* and *SI Appendix*, Fig. S3 *A*–*C*). In split-root systems, fine LR lengths and proportions were reduced on the mycorrhizal side, regardless of CO_2_ levels (Fig. 2 *E* and *F*). The tendency toward reduced fine LR lengths and proportion was nonsignificant in *pt11* mutants under aCO_2_ and eCO_2_ levels (Fig. 2 *E* and *F*), suggesting that *PT11* contributes significantly to this local suppression of fine LR growth; but other independent mechanisms possibly exist. In contrast, CRs and large LRs lengths were largely insensitive to local mycorrhizal status or CO_2_ levels (*SI Appendix*, Fig. S3 *B* and *C*). Nonetheless, as *pt11* is still partially colonized, we cannot exclude the possibility for a threshold of fungal colonization and/or nutrient exchange to achieve local suppression of fine LR development.

### Radiotracer Experiments Reveal That Suppression of Direct Uptake is a Local Response Occurring Only After Symbiotic Pi Uptake.

To directly trace the Pi uptake pathway in the split-root setup, isotope-labeling was performed, whereby ^33^P was supplied to the nonmycorrhizal compartment (Root A) to track direct uptake, while ^32^P was supplied to the AM compartment (Root B) via a hyphal-only chamber to track symbiotic uptake. This differential labeling set up allowed simultaneous monitoring of both direct (nonsymbiotic) and indirect (symbiotic) uptake of P in the same plant (Fig. 2*A*).

In mycorrhizal set ups, most of ^32^P taken up in the AM compartment is expected to be mediated by known symbiotic Pi transporters, *PT11* and *PT13* that appear exclusively expressed in that compartment (29), then translocated to the shoots where most of it exists, and subsequently a small fraction is allocated to the NM compartment. Indeed, presence of AM significantly increased ^32^P levels in the AM compartment (Root B; Fig. 2*I*), shoots (Fig. 2*G*), and also NM compartment (Root A; Fig. 2*H*); and the amount in all three compartments further increased with eCO_2_, indicating that the increased AM colonization comes with increased symbiotic function at the level of symbiotic P uptake. Low but detectable transfer of ^32^P could be observed in *pt11* mutants, and this too increased with exposure to elevated CO_2_, but was overall not statistically significant in either shoot, root B, or total ^32^P pools (Fig. 2 *G*–*I* and *SI Appendix*, Fig. S3*G* respectively). This residual uptake activity could be mediated by PT13, another symbiotic PHT1 transporter expressed in the AM compartments of both WT and *pt11* (*SI Appendix*, Fig. S3*M*). As quality control, ^32^P levels in NM/NM (fully noncolonized) controls reflected very low background leakage or systemic redistribution of ^32^P in the root compartment where fungal hyphae was not provided, and this slightly increased, albeit insignificantly, in eCO_2_ (Fig. 2 *G*–*I*).

We then examined the direct P uptake pathway by tracing ^33^P, which was taken up in the NM compartment (Root A). Most (>90%) of the ^33^P taken up by the plant is stored in the shoot (Fig. 2*J* and *SI Appendix*, Fig. S3 *D* and *E*), and a fraction is reallocated to Root B (10 to 15%, Fig. 2*L* and *SI Appendix*, Fig. S3*F*), where ^33^P was not provided. At aCO_2_, ^33^P uptake by the NM roots (root A) was generally similar in noncolonized WT, colonized WT, and *pt11* (Fig. 2*K*). ^33^P content in *pt11* mutants under elevated CO_2_ was slightly reduced (Fig. 2*K*), but not the amount in shoots and the total pool (Fig. 2*J* and *SI Appendix*, Fig. S3*D*). We furnished our observations with gene expression analyses, observing limited differences in expression of two well-characterized direct uptake transporters, *PT2 and PT6* (*SI Appendix*, Fig. S3 *J* and *K*). Expression of *PT2*, *PT6* remained high in all NM (Root A) compartments, suggesting limited differences in direct uptake activities under ambient and elevated CO_2_ levels (*SI Appendix*, Fig. S3 *J* and *K*). In the split-root mycorrhizal setups, expression of *PT2* and *PT6* remained high in the NM compartment (Root A); but in wild-type plants where colonization is established to high levels in the AM compartment (Root B), significant suppression of *PT2* occurs (*SI Appendix*, Fig. S3*J*), with the same tendency for *PT6*. This suppression therefore occurs locally, and requires functional symbiotic P exchange, as expression of *PT2* and *PT6* remained high in the mycorrhizal compartment of *pt11* roots (*SI Appendix*, Fig. S3 *J* and *K*). Taken together, suppression of direct P uptake pathway occurs at a local level, only when symbiotic P is acquired via AM fungi.

Altogether, the data demonstrate that under elevated CO_2_, there is increased direct and indirect uptake of the labeled ^32/33^P species in their respective compartments, as well as increased P mobilization to the reciprocal compartment. The data are also consistent with the expectation that rice PT11 is the major symbiotic Pi transporter and in its absence, most (c.90%) of ^32^P uptake is compromised (29) and the plant continues to rely on direct uptake transporters. Gene expression of *PT2*, *PT6* for direct uptake did not show significant increase from ambient to elevated CO_2_, indicating that the increase in P uptake could be a result of transporter activity over time, or mediated by other *PTs* not profiled here. Most importantly, the split-root experiment and radiotracer data both reveal that during symbiosis, inhibition of direct uptake of P occurs at a local level as a strong suppression of *PT2,* and partial suppression of *PT6* only occur in the AM compartment (*SI Appendix*, Fig. S3 *J* and *K*), and require functional nutrient exchange via PT11 to drive this inhibition; while correspondingly ^33^P uptake in the NM compartment is not affected.

### Mapping Physiological Responses to the Root Transcriptome.

**Fig. 3.**
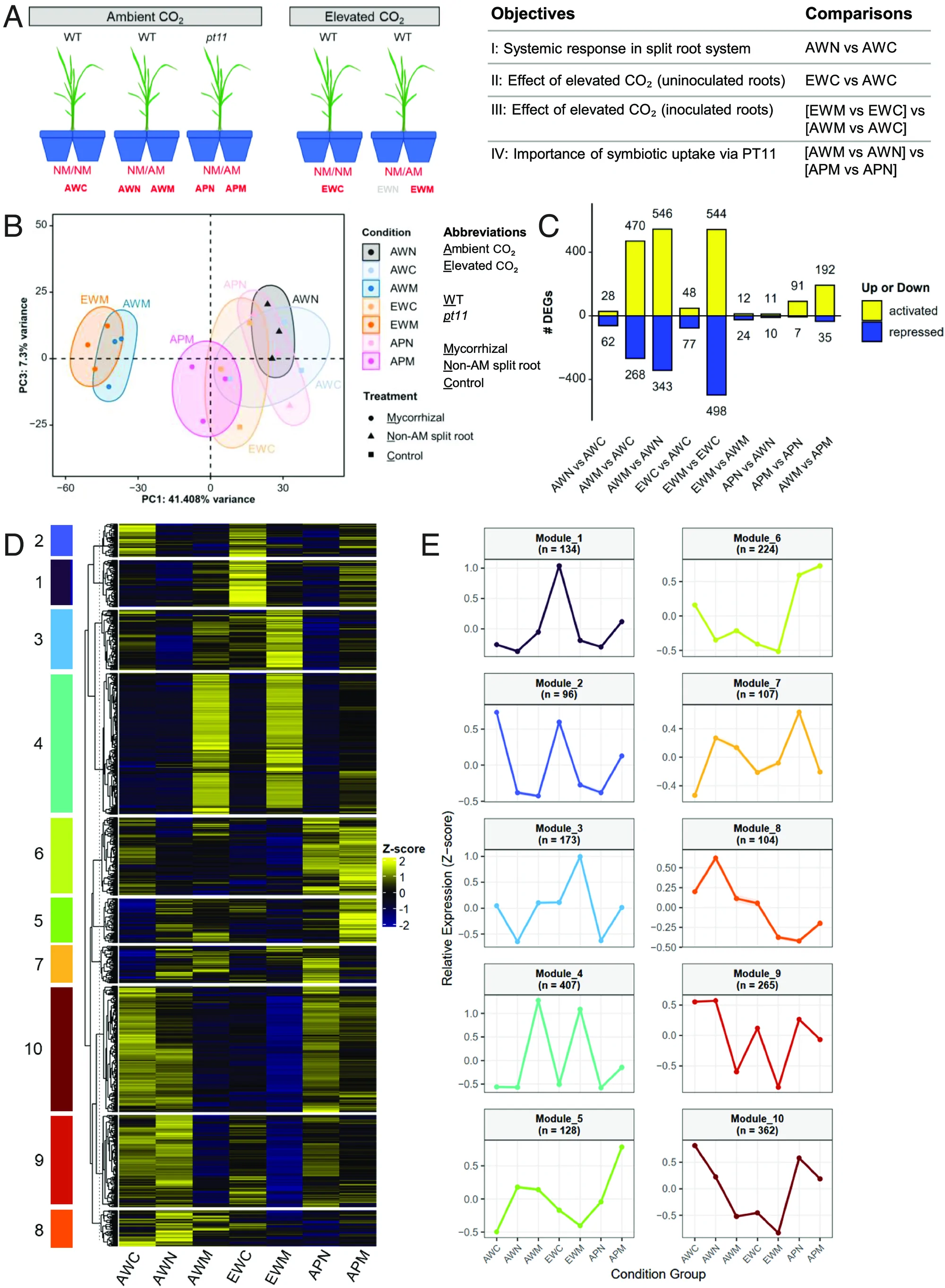
Experimental design and transcriptomic responses of plants to elevated CO_2_ and arbuscular mycorrhizal (AM) symbiosis. (*A*) Schematic of the experimental design showing plants grown under ambient or elevated CO_2_ with three root treatments: arbuscular mycorrhizal (AM), non-AM split-, and nonmycorrhizal control. Gray labels indicate conditions included in the experimental design but not subjected to RNA-seq analysis. (*B*) Principal component analysis (PCA) of RNA-seq expression profiles. Points represent individual biological replicates, colored by experimental condition. Shapes denote treatment (AM, non-AM split-root, or control). Percent variance explained by each principal component is indicated on the axes. Abbreviations used in the sample names specified in the key. (*C*) Numbers of differentially expressed genes (DEGs) for pairwise contrasts among CO_2_ and mycorrhizal treatments. Yellow and blue bars indicate up-/down-regulated genes respectively. Numbers denote DEG counts (false discovery rate, FDR–adjusted *P* < 0.05). (*D*) Heatmap of z-score–normalized expression values for genes grouped by weighted gene coexpression network analysis (WGCNA) modules. Rows represent individual genes and columns represent experimental conditions. Module color assignments are shown along the left margin. (*E*) Eigengene expression profiles for selected coexpression modules across experimental conditions. Values represent standardized module z-scores, illustrating coordinated transcriptional responses to CO_2_ level and mycorrhizal status. Experimental conditions are ordered consistently across panels. Module colors assigned in (*D*) correspond to the individual coexpression module profiles shown in (*E*).

Our physiological analyses revealed that eCO_2_ alters plant growth, root architecture, and Pi acquisition through coordinated local and systemic regulation of direct and symbiotic uptake pathways. While these measurements define the functional outcomes of eCO_2_, they do not reveal the underlying molecular mechanisms coordinating these spatially resolved responses. To identify the transcriptional programs associated with these physiological changes, we profiled the root transcriptome in a split-root experiment comprising wild-type plants grown under aCO_2_ and eCO_2_, and *pt11* mutants grown under aCO_2_ (Fig. 3*A*).

Global transcriptional responses were assessed across the groups of samples by principal component analysis (PCA, Fig. 3*B*). Samples separated predominantly along PC1 (41.4% of variance), which clearly distinguished wild-type mycorrhizal roots from nonmycorrhizal roots. Notably, *pt11* mycorrhizal samples clustered closer to nonmycorrhizal than to wild-type mycorrhizal samples, consistent with reduced activation of the full symbiotic transcriptional program in the mutant. By contrast, separation associated with eCO_2_ was comparatively modest, indicating that CO_2_ treatment had a weaker effect on global root transcriptome than AM colonization or loss of PT11-dependent symbiotic function.

Consistent with the PCA, differential expression analysis revealed extensive transcriptional reprogramming associated with AM colonization in wild-type roots, with approximately 1,000 differentially expressed genes (DEGs) in colonized roots relative to nonmycorrhizal controls under both aCO_2_ and eCO_2_ conditions (AWM vs. AWC; EWM vs. EWC, Fig. 3*C*). By contrast, noncolonized halves of split-root systems exhibited only minor transcriptional changes compared to fully nonmycorrhizal controls (AWN vs. AWC; 90 DEGs, Fig. 3*C*), indicating subtle systemic transcriptional signaling to noncolonized roots. Comparisons between aCO_2_ and eCO_2_ conditions yielded relatively few DEGs in both mycorrhizal (EWM vs. AWM; 36 genes) and nonmycorrhizal roots (EWC vs. AWC; 125 DEGs), reinforcing the notion that eCO_2_ elicits limited transcriptional reprogramming (Fig. 3*C*).

In *pt11* mutants, colonized root compartments at aCO_2_ (APM) showed a reduced number of differentially expressed genes (APM vs. APN; 98 DEGs) relative to wild-type mycorrhizal roots, while noncolonized compartments (APN) remained transcriptionally similar to controls (APN vs. AWN; 21 DEGs; Fig. 3*C*). Together, these analyses demonstrate that AM colonization is the primary determinant of root transcriptome structure, whereas eCO_2_ and systemic signaling exert comparatively mild effects at the transcriptional level (16).

To complement the DEG analyses, we performed weighted gene coexpression network analysis on the top 2,000 genes with variable expression, identifying 10 distinct modules of genes with synchronized expression patterns (Fig. 3*D*), providing a holistic view of the transcriptional changes across all seven conditions. These modules capture major regulatory trends associated with AM colonization, systemic signaling, eCO_2_, and misregulation in *pt11*. Modules 1 and 2 comprise genes repressed by systemic signaling from colonized root halves but induced in noncolonized roots under eCO_2_ and in colonized *pt11* roots, hinting at a putative defense-related signature. Modules 3 and 4 contain genes strongly induced by AM colonization, with Module 4 in particular going from low expression in uncolonized roots to high expression in the colonized roots. Conversely, Modules 9 and 10 represent genes broadly suppressed during symbiosis, with stronger repression under eCO_2_ and partial derepression in *pt11* mutants; Module 10 genes are additionally suppressed by systemic signaling. Module 5 includes genes induced by eCO_2_, systemic signaling, or colonization under ambient CO_2_, but notably not in colonized roots under eCO_2_. Module 6 comprises genes suppressed by either colonization or systemic signaling but misregulated in *pt11* mutants. Module 7 contains genes induced by systemic signaling, colonization, and eCO_2_, whereas Module 8 includes genes induced by systemic signaling and weakly in colonized *pt11* roots but repressed by colonization under eCO_2_, again suggesting a defense-associated response. Together, this network analysis provides an integrated framework linking physiological outcomes with transcriptional coordination across local and systemic root responses.

#### I: Defining global and systemic responses to AM colonization.

We first sought to define the systemic responses to AM colonization, that is, genes differentially expressed in the noncolonized sections of a mycorrhizal plant (AWN vs. AWC). Whereas AM symbiosis is known to elicit systemic signaling in shoots and roots, studies on corresponding transcriptional responses remains limited. An early microarray-based study by Liu et al. identified only three *Medicago truncatula* genes induced in the noncolonized half of a split-root system (35). Consistent with this, our physiological analyses detected no differences in root architecture or Pi uptake between noncolonized compartments of mycorrhizal plants and fully nonmycorrhizal controls. Transcriptomic profiling likewise revealed a weak systemic response, with only 90 differentially expressed genes (logFC > 1, *P*-adjusted < 0.05) between AWN and AWC (*SI Appendix*, Fig. S4*A*). Of these 90 genes, 28 were upregulated and 62 were downregulated, and canonical symbiosis-associated genes remained unchanged, indicating the absence of local mycorrhizal signaling (*SI Appendix*, Figs. S4*A* and S5). Notably, 14 genes (16%) were regulated in the same direction in colonized roots (AWM vs. AWC), suggesting that the noncolonized compartment adopts a partially primed transcriptional state in anticipation of fungal invasion (*SI Appendix*, Figs. S4 *C* and *F* and S5).

Functionally, DEGs in AWN relative to AWC were predominantly downregulated and included gene members for specialized metabolism and defense-related processes. These included biosynthetic gene clusters involved in diterpenoid (momilactone) biosynthesis (36), jasmonate-linked lipid metabolism, flavonoid and ethylene biosynthesis, as well as multiple pathogenesis-related protein classes, *PR1, PR4, PR5, PR10*. Genes implicated in defense signaling were also downregulated (*SI Appendix*, Fig. S4*A*), suggesting a systemic attenuation of defense responses in noncolonized roots of mycorrhized plants. By contrast, fewer genes were significantly induced, including a basic helix–loop–helix transcription factor (*bHLH166*), which was also induced in mycorrhizal roots, genes associated with cell wall remodeling and metabolism (*AUX5*, *BAMY*), as well as two *NPH3* orthologs (*SI Appendix*, Figs. S4 *A* and *F* and S5). In *Arabidopsis*, *NPH3* functions as a substrate adaptor in an E3 ubiquitin ligase complex involved in blue-light signaling, suggesting a role in signal integration rather than direct defense or nutrient acquisition in these root sectors.

Together, these data provide a comprehensive glimpse of AM-induced systemic signaling in mycorrhizal plant roots. Gene expression changes indicate that systemic transcriptional responses in noncolonized roots are dominated by repression of chemical and protein-based defense pathways, accompanied by limited induction of signaling and cell wall–associated genes. This transcriptional state reflects a primed root compartment that anticipates symbiotic accommodation while leaving core nutrient acquisition pathways largely unchanged.

#### II: Defining the transcriptional response to elevated CO_2_ reveals a convergent transcriptome favoring symbiosis.

Physiological analyses showed that eCO_2_ enhanced levels of AM colonization in rice and promoted biomass, root architecture remodeling, and Pi uptake via both direct and symbiotic uptake pathways. To determine whether these effects are associated with transcriptional reprogramming that promotes AM symbiosis, we compared the transcriptome of nonmycorrhizal control roots under eCO_2_ (EWC) vs. aCO_2_, (AWC), minimizing confounding systemic effects. Elevated CO_2_ elicited a modest transcriptional response, with 125 DEGs (48 upregulated, 77 downregulated, Fig. 3*C*), consistent with attenuated CO_2_ responses reported under nitrogen- and iron-replete conditions for *Arabidopsis thaliana* (16).

Because eCO_2_ increased AM colonization, particularly at higher P availability, we next asked whether this response reflects an enhanced symbiotic competence in nonmycorrhizal roots. Recent studies have shown that permissive signaling for AM symbiosis is regulated by the D14L/SMAX1 module (37), the P starvation regulators PHR1/2/3 (38, 39), and the GRAS transcription factors NSP1/2 (40, 41); which collectively control the expression of symbiosis signaling components across multiple stages of the interaction. However, overlap between CO_2_-responsive genes and published SMAX1- or PHR-regulated gene sets was minimal, comprising only 12 genes (~10% of the CO_2_-responsive transcriptome, *SI Appendix*, Fig. S4 *D* and *E*), most of which do not have known roles in AM symbiosis (*SI Appendix*, Fig. S4*G*). These results argue against transcriptional activation of established symbiosis-permissive networks under eCO_2_. Even when the analysis was relaxed to include genes significant at the nominal *P*-value and focused specifically on symbiosis signaling components, the overlap remained limited to six genes, including *D10*-*like/CCD8a* and *D10/CCD8b* (*SI Appendix*, Fig. S4*G*), two key enzymes in strigolactone biosynthesis (42). This limited overlap is consistent with expectations that eCO_2_ may enhance strigolactone production based on prior work in *Arabidopsis* and tomato (43), although strigolactone levels were not directly measured in this study.

Instead, the loss of CO_2_-induced increases in colonization in *pt11* mutants suggests that enhanced colonization under eCO_2_ depends on functional symbiotic nutrient uptake rather than a transcriptionally more permissive state before symbiosis. Because eCO_2_ is known to promote photosynthetic carbon fixation and biomass accumulation in C_3_ plants, these observations are consistent with increased carbon availability contributing to intensified symbiotic activity. However, carbon allocation and fungal metabolic activity were not directly measured in this study.

We therefore examined the asymbiotic CO_2_-responsive transcriptome in an unbiased manner. Elevated CO_2_ induced genes associated with carbohydrate metabolism and root growth, including *Neutral Invertase 8* (*NIN8*), in line with observed increases in root biomass and architectural remodeling (44). By contrast, a suite of fifteen genes involved in iron uptake, homeostasis, and iron deficiency signaling (*IRT1*, *IRT2*, *NRAMP1*, *IMA1*, among others; *SI Appendix*, Fig. S6) was downregulated, indicating attenuation of iron starvation responses (reviewed in ref. 45). Because suppression of iron deficiency signaling also occurs during AM colonization (*SI Appendix*, Fig. S6*B*, AWM/AWC), we hypothesize that this transcriptional shift may further promote a root environment conducive to growth and symbiosis.

Since eCO_2_ did not strongly activate canonical symbiosis signaling pathways, we next asked whether the asymbiotic CO_2_-responsive transcriptome nevertheless showed partial convergence toward AM-associated transcriptional states. Indeed, eCO_2_ partially shifted the transcriptome toward an AM-like expression profile: six genes were commonly upregulated and 29 downregulated between CO_2_- and AM-responsive gene sets, with no genes regulated in opposite directions. This accounts for 28% of the CO_2_ response and accords with Modules 3 and 7. Notably, several shared induced genes—including an *NPH3* ortholog and *bHLH166*—were also systemically induced in split-root experiments, demonstrating convergence between CO_2_- and symbiosis-associated transcriptional programs (*SI Appendix*, Fig. S4 *C* and *F*). In parallel, eCO_2_ also induced *PT3* and *PT9*, two less-characterized *PHT1* transporters (*SI Appendix*, Fig. S7). This is consistent with radiotracer data showing enhanced direct Pi uptake under eCO_2_ and highlights the value of the comprehensive transcriptome profiling undertaken here.

Together, these data suggest that eCO_2_ enhances Pi acquisition capacity primarily by increasing direct Pi uptake, altering carbon metabolism, suppressing iron starvation, while simultaneously creating a transcriptional environment that favors AM colonization through mechanisms distinct from canonical symbiosis signaling pathways.

#### III: Defining the mycorrhizal transcriptome under eCO_2_.

The absence of enhanced symbiotic signaling genes under eCO_2_ prompted us to examine transcriptional responses within mycorrhizal root compartments under aCO_2_ and eCO_2_, to identify molecular processes underlying increased colonization, enhanced symbiotic nutrient capture, and suppression of fine LR development. Establishment of AM symbiosis triggered extensive transcriptional reprogramming in colonized roots. Under aCO_2_, AM colonization (AWM vs. AWC) resulted in 738 DEGs (420 up, 268 down, Fig. 3*C*), whereas under eCO_2_ a similarly extensive but transcriptionally broader response was observed (EWM vs. EWC; 1,042 DEGs, 544 up and 498 down, Fig. 3*C*). This enhanced response might be the result of higher levels of mycorrhizal colonization under eCO_2_.

**Fig. 4.**
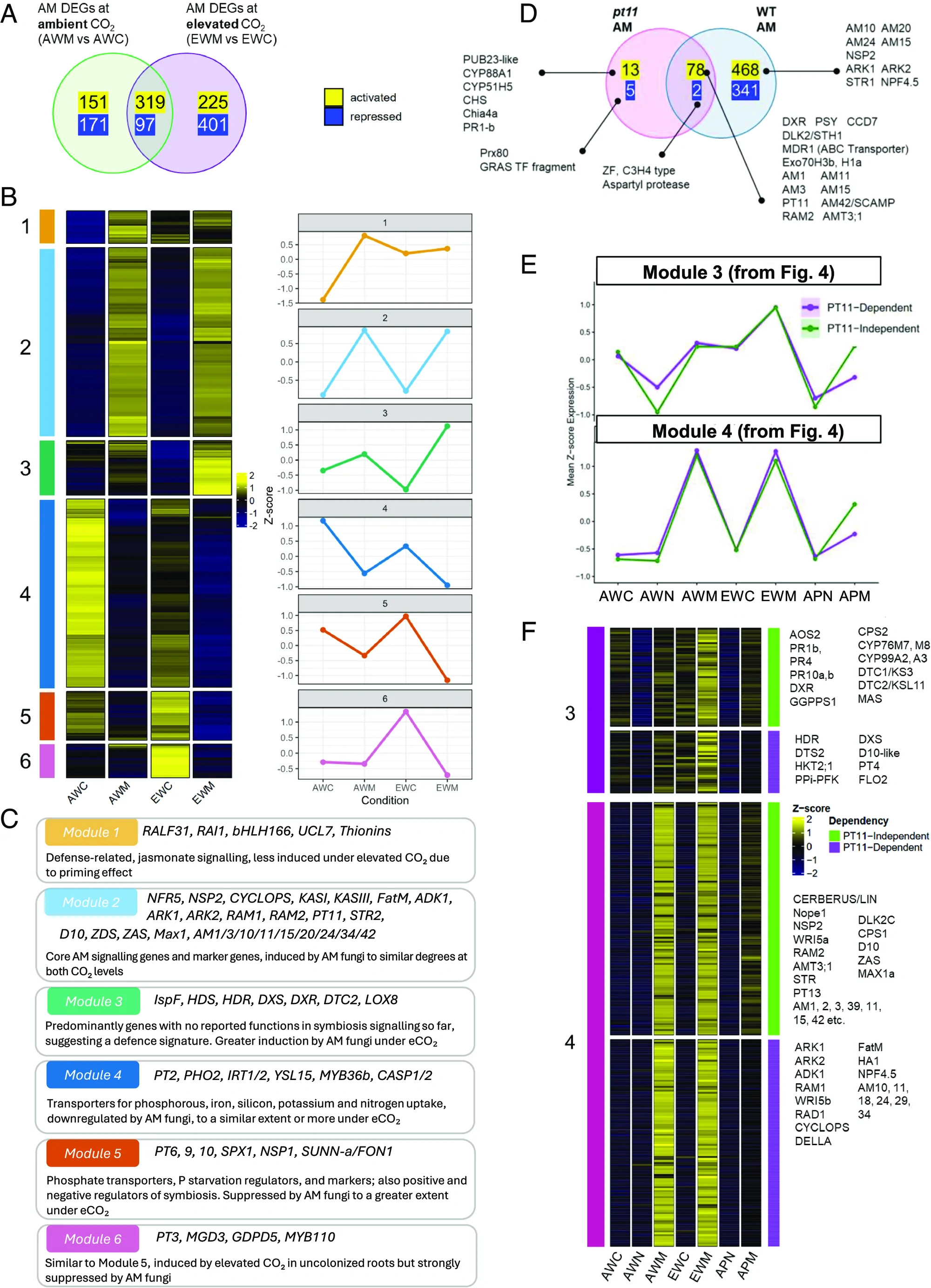
CO_2_-dependent remodeling of the mycorrhizal transcriptome and PT11-dependent transcriptional programs. (*A*) Venn diagrams showing overlap between genes differentially regulated by AM symbiosis under ambient vs. elevated CO_2_, highlighting shared and condition-specific transcriptional responses. Numbers in yellow and blue indicate up- and down-regulated genes, respectively. (*B*) Heatmap showing z-score–normalized expression of differentially expressed genes (DEGs) identified from mycorrhizal roots under ambient and elevated CO_2_ conditions. Genes are grouped into six coexpression modules based on hierarchical clustering. Columns represent experimental conditions. See Fig. 3 for definitions to the abbreviations. Module eigengene profiles summarizing the mean expression pattern of each module across conditions. (*C*) Module behavior, gene membership, and predicted significance. See *SI Appendix*, Fig. S7 for expression profiles of highlighted genes and *SI Appendix, Supplemental Note 1* for expanded description. (*D*) Venn diagrams showing overlap between genes induced by AM in wild-type vs. *pt11* mutants. See *SI Appendix*, Fig. S8 for full list of misregulated genes in *pt11.* (*E*) Mean z-score expression profiles for representative modules (Modules 3 and 4, from Fig. 3), separated into PT11-dependent and PT11-independent subsets across all experimental conditions. (*F*) Heatmap of AM-induced genes further classified according to PT11 dependency. Gene blocks are labeled as PT11-independent (green) or PT11-dependent (purple) based on their induction in wild-type vs. *pt11* mutant mycorrhizal roots.

Comparison of the two abovementioned AM-induced DEG sets under aCO_2_ and eCO_2_ conditions revealed only partial overlap, with 319 commonly induced genes (45.8% of all induced genes) and 97 commonly repressed genes (14.5% of all repressed genes; Fig. 4*A*), arguing for distinct transcriptional programs being engaged, depending on CO_2_ availability. To capture these condition-dependent responses, we combined DEGs from both comparisons (1,364 genes total; 695 up, 669 down) and performed clustering analysis, which resolved six expression modules with distinct regulatory profiles (represented in Fig. 4*B*, described in Fig. 4*C* and detailed in *SI Appendix*, Fig. S7 and *Supplemental Note 1*).

A core AM transcriptional program (Module 2) was robustly induced regardless of CO_2_ level, comprising canonical symbiosis markers (*AM1*, *AM3*, *ARK1*, *RAM1*, *FatM*, *PT11*, *PT13*, *NPF4.5*) strictly in colonized roots, in line with published promoter activities (29, 46–53). Elevated CO_2_ modified this program in two directions: It primed defense-associated and jasmonate-linked genes before colonization (Module 1), while specifically amplifying specialized metabolism, notably diterpenoid phytoalexin (momilactone), within colonized roots (Module 3), raising the possibility of regulated defense activation linked to arbuscule turnover. In parallel, AM symbiosis broadly repressed direct nutrient acquisition, including Pi transporters, iron and silicon transporters, purple acid phosphatases, and strikingly, genes involved in Casparian strip formation [*MYB36b*, *CASP1*, *CASP2*; Module 4, (54, 55)], suggesting remodeling of endodermal diffusion barriers. Under eCO_2_, this suppression extended to additional *PHT1* transporters, nitrogen acquisition genes, Pi starvation markers, and *SUNN-a/FON1*—a CLAVATA-clade receptor implicated in negative autoregulation of colonization [(56–58), Modules 5 and 6].

#### IV: PT11-dependent transcriptional programs during AM symbiosis.

Rice *PT11* mediates symbiotic Pi uptake in a nonredundant manner with *PT13* (29, 30). Physiological analyses showed that PT11 is required for local Pi uptake at arbuscules during AM symbiosis and for the associated suppression of direct Pi uptake. This effect was restricted to colonized root compartments, demonstrating that symbiotic Pi uptake is required locally to initiate downstream signaling responses.

To define transcriptional programs dependent on PT11, and misregulation associated with defective symbiotic nutrient transfer, we compared colonized and noncolonized compartments of split-root *pt11* plants (APM vs. APN). Colonized *pt11* roots retained a small core AM transcriptional programme, with 80 genes overlapping with the WT response (Fig. 4*D*). However, the majority of WT AM-induced genes (>800 genes) failed to be activated in *pt11*, indicating that PT11-dependent nutrient exchange is required for full progression of the symbiotic transcriptional programme.

To further resolve PT11-dependent vs. PT11-independent transcriptional responses agnostic to DEG cutoff, we examined expression profiles of AM-induced genes from Modules 3 and 4 of Fig. 4, in *pt11* roots. These genes segregated into two groups: one showing robust induction in *pt11* (PT11-independent) and another showing weak or absent induction (PT11-dependent), corresponding to distinct intersections in the DEG overlap analysis (Fig. 4 *E* and *F*). Consistent with the reduced colonization and stunted arbuscule development previously reported for *pt11* and orthologous mutants in *Medicago* (29, 32, 59), PT11-independent genes largely comprised early symbiosis signaling and accommodation components—e.g., *NSP2*, *NOPE1*, *WRI5a,* and a suite of AM marker genes, as well as some symbiotic nutrient transporters *AMT3;1*, *STR*, and *PT13*. In parallel, specialized metabolism pathways—particularly jasmonate, momilactone, and strigolactone—were also activated independent of PT11 (Module 3, Fig. 4*F*).

By contrast, a second set of induced genes was largely absent in *pt11* roots and appeared to require functional PT11 and/or effective nutrient exchange. This PT11-dependent group (Fig. 4 *E* and *F*) included signaling components (*ARK1*, *ARK2*, *CYCLOPS*, *DELLA*, *ADK1*, *WRI5b*), metabolic genes (*FatM*), proton pump *HA1*, and diterpenoid biosynthesis genes (*HDR*, *DTS*, *DXS*). The failure to activate this transcriptional wave coincided with, and provides functional explanation for impaired arbuscule maturation, reduced symbiotic nutrient transfer and reduced overall colonization observed in *pt11*.

Finally, analysis of genes uniquely misregulated in *pt11* revealed activation of genes not induced in wild-type mycorrhizal roots. These included two glucose-6-phosphate translocators (*GPTs*), *AGPS1*, and *GBSSII* (starch synthase), suggesting diversion of carbon flux toward starch biosynthesis (*SI Appendix*, Fig. S8). Several defense-associated genes, including β-expansins, *PR1b*, and a jasmonate-induced chitinase, were also upregulated (Fig. 4*D* and *SI Appendix*, Fig. S8). And a substantial fraction of misregulated genes lacked functional annotation (*SI Appendix*, Fig. S8).

### Elevated CO2 imposes stronger transcriptional shutdown of the direct phosphorous uptake pathway.

Finally, given our focus on phosphorus acquisition, we examined the expression profiles of Pi transporters and key regulators of P signaling and homeostasis across treatments (*SI Appendix*, Fig. S9). Modules 4, 5, 6 of Fig. 4 *B* and *C* already suggested differential regulation of P homeostasis under aCO_2_ and eCO_2_, but a P-focused analysis allowed a more comprehensive view.

Under aCO_2_, AM colonization repressed only two members of the *PHT1* family (*PT2* and *PT3;* AWM/AWC, *SI Appendix*, Fig. S9*B*). Under eCO_2_, AM colonization suppressed four additional *PHT1* transporters (*PT6, PT8b*, *PT9, PT10*; EWM vs. EWC, *SI Appendix*, Fig. S9*B*), indicating a broader downregulation of direct Pi uptake pathways. This extended to downregulation of vacuolar Pi transporters (*SPX-MFS2* and *VPE1*), whereas transporters associated with mitochondrial and plastidial phosphate flux (*PHT3* and *PHT4*) remained comparatively stable across conditions. Expression of *PHO1*, which mediates xylem loading of phosphate showed a tendency toward repression during AM symbiosis under both CO_2_ regimes (*SI Appendix*, Fig. S9*B*).

Genes involved in mobilization of organic phosphate for direct uptake were likewise downregulated in colonized roots under both aCO_2_ and eCO_2_ levels (AWM vs. AWC and EWM vs. EWC, *SI Appendix*, Fig. S7*B*), represented by purple acid phosphatases (*PAP10c* and *PAP21b*). In parallel, regulators of P signaling were repressed under eCO_2_ and AM symbiosis: *SPX1*, an inositol pyrophosphate sensor that represses PHR transcription factor activity, was downregulated in mycorrhizal roots under eCO_2_, as was *NIGT1*, a transcriptional repressor that integrates nitrogen and P signaling (EWM vs. EWC, *SI Appendix*, Fig. S9*B*).

Consistent with improved P nutritional status, P starvation response markers (PSR: *IPS1*, *SQD2.2*, *NGD3*, *GDPD5*) were further suppressed in mycorrhizal roots under eCO_2_ (EWM vs. EWC, *SI Appendix*, Fig. S9*B*). Inspection of the VST-normalized expression profiles (*SI Appendix*, Fig. S9*A*) indicates that this broader repression partly reflects moderately higher baseline expression of Pi uptake and P starvation-response genes in nonmycorrhizal roots under eCO_2_, followed by their suppression during AM symbiosis, rather than uniformly lower absolute expression in mycorrhizal roots. Thus, eCO_2_ appears to increase the dynamic range of AM-associated repression for several P-responsive genes.

Together, this P-focused analysis indicates that while eCO_2_ alone can induce a subset of *PHT1* transporters), the most extensive and coordinated remodeling of Pi transport and storage occurs during AM symbiosis under eCO_2_ (EWM vs. EWC, *SI Appendix*, Fig. S9). Notably, all of these transcriptional changes are abolished in *pt11* mutants (APM vs. APN, *SI Appendix*, Fig. S9), consistent with physiological and radiotracer experiments. These observations solidify the critical role of PT11-mediated transport, and the downstream events it engages, in reinforcing and favoring a symbiotic transcriptome over the direct Pi uptake pathway. Interestingly, in mycorrhizal wild-type plants and especially under eCO_2_; despite repression of direct uptake and storage pathways, overall P status improved and P starvation signaling is attenuated, indicating effective compensation through symbiotic uptake and redistribution.

## Discussion

Our work demonstrates that eCO_2_ modulates Pi acquisition and AM symbiosis in rice primarily by increasing carbon availability and reinforcing functional symbiotic Pi acquisition, rather than by activating canonical host permissive signaling pathways. Across physiological, radiotracer, and transcriptomic analyses, a consistent pattern emerges in which successful nutrient uptake through the symbiotic Pi transporter PT11 acts at a *local* level to coordinate root architectural remodeling, suppression of direct Pi uptake, and progression of symbiotic transcriptional programs. These findings suggest functional symbiotic Pi transport via PT11 contributes to progression of symbiotic developmental and transcriptional programs. (31, 33).

Elevated CO_2_ increased plant biomass and total P accumulation but these gains were not amplified by AM symbiosis, reflecting the carbon cost of symbiosis. Instead, the principal benefit of AM symbiosis under low phosphate corresponded to improved Pi uptake efficiency, allowing plants to achieve comparable shoot P content with reduced root investment. Root architectural responses support this shift: Fine lateral root development was locally suppressed in colonized compartments, consistent with reduced reliance on elongation-based foraging when symbiotic uptake is active. The partial persistence of this response in *pt11* indicates that both Pi uptake-dependent and independent mechanisms contribute to local architectural control. This adds a hitherto unknown spatial and signaling context to root architecture remodeling in rice, for which the signaling molecules, receptors, and components mediating this response have been progressively revealed (27, 60, 61). We note that this growth benefit was not evident in the whole-pot experiment at the equivalent P supply (P20; Fig. 1 *C* and *D*), where AM plants instead accumulated less overall biomass than NM plants. Such context dependency in mycorrhizal growth response is an unresolved feature (62) and does not affect the mechanistic conclusions drawn here, which rest on the paired, within-plant comparisons of the split-root experiment (Fig. 2).

Dual-isotope tracing informed that direct and symbiotic Pi uptake pathways are tightly coordinated but spatially separated. Suppression of direct uptake occurs locally in colonized roots and requires functional symbiotic Pi transfer, whereas noncolonized roots retain direct uptake capacity. Elevated CO_2_ enhanced both direct and symbiotic Pi fluxes without significantly increasing expression of major Pi transporters, indicating that increased uptake reflects sustained transporter activity over a larger root system and over time, likely driven by greater carbon availability and root growth rather than transcriptional induction. The relatively modest transcriptional response to eCO_2_ despite pronounced physiological effects suggests that many of these responses may be regulated through cumulative transporter activity, spatially restricted transcriptional changes, or posttranscriptional mechanisms not fully captured by bulk root transcriptome profiling.

At the transcriptional level, AM colonization overwhelmingly dominated root gene expression, whereas eCO_2_ alone led to relatively modest changes. Importantly, eCO_2_ did not substantially activate known permissive symbiosis networks controlled by *SMAX1, PHR*, or *NSP* transcription factors. Instead, *pt11* mutants abolished the CO_2_-dependent increase in colonization. This points to carbon supply, functional nutrient exchange and defense changes, rather than host signaling competence, as the primary drivers of enhanced symbiosis under eCO_2_. This also offers a compelling explanation for differential effects of eCO_2_ on the level of symbiotic engagement across different species tested, as the effect is not wired through a canonical symbiosis signaling pathway.

Elevated CO_2_ nonetheless altered the qualitative state of the mycorrhizal transcriptome. Mycorrhizal roots under eCO_2_ displayed strong induction of specialized metabolism, including jasmonate-linked pathways and diterpenoid phytoalexin biosynthesis, alongside repression of nutrient transporters (notably iron), P starvation responses, and Casparian strip–associated genes. These changes suggest a reconfiguration of root defense chemistry, nutrient routing, and barrier properties during symbiosis when carbon supply is abundant. Whether these defense-associated responses function to constrain fungal proliferation, regulate arbuscule turnover, or spatially compartmentalize symbiotic interfaces remains to be resolved. It is currently unclear, especially in rice, how iron signaling affects AM symbiosis. Iron deficiency suppresses AM symbiosis in tomato (63) but promotes symbiosis (under replete P) in Petunia (64), both Solanaceous species. Moreover, the importance of iron is further suggested by the observations that iron deficiency regulates nodulation symbiosis signaling through *NSP1* (65, 66) and that in rice the iron sensor HRZs ubiquitinates and degrades PHR2 (67), a key regulator of P starvation and symbiosis. It is tempting to speculate that the downregulated iron signature (of transporters, transcriptional regulators, and starvation markers), opens a new vista of symbiosis signaling.

Analysis of *pt11* mutant further reveals that AM symbiosis proceeds through at least two transcriptional phases. An initial, PT11-independent phase activates early signaling and accommodation programs, whereas a second PT11-dependent phase reinforces signaling networks, lipid and secondary metabolism, and proton pumping required for sustained nutrient exchange. Failure to activate this second phase coincides with impaired arbuscule maturation, altered carbon partitioning toward starch accumulation, and activation of defense responses, highlighting symbiotic Pi uptake as a key driver of transcriptional progression rather than a downstream consequence of symbiosis. This provides additional evidence to support the postulated “transcriptional waves” that occur during symbiosis (68, 69).

Finally, a P-centric analysis shows that elevated CO_2_ intensifies AM-associated suppression of direct Pi uptake and storage pathways while overall plant P status improves, accompanied by an attenuated P starvation signaling. This indicates effective compensation via symbiotic Pi uptake and redistribution, even as direct pathways are transcriptionally repressed. The persistence of strong repression of nonsymbiotic Pi transporters despite reduced expression of known regulators such as SPX1, SPX5, and NIGT1 (39, 70–72) suggests additional spatially acting control mechanisms that remain to be identified.

Together, these findings predict that rising atmospheric CO_2_ will shift rice, when in the mycorrhizal state, toward greater reliance on symbiotic Pi acquisition by enhancing carbon availability and reinforcing nutrient-exchange checkpoints at the arbuscule. By identifying PT11-mediated Pi uptake as a key regulator of root architecture, uptake pathway choice, and transcriptional progression during symbiosis, this work reframes symbiotic nutrient transport as an active control point rather than an accompanying passive outcome.

Because this study was conducted in the model rice cultivar Nipponbare, the extent to which these responses generalize across rice genotypes remains to be established. Given the well-documented variability in plant growth responses to AM fungi across experimental systems, future comparative studies spanning diverse host genotypes, symbiotic systems, and environmental conditions will be important to determine how broadly eCO₂ reinforces symbiotic Pi uptake and reshapes root nutrient acquisition strategies. Nonetheless, these insights highlight symbiotic capture capacity and carbon allocation as critical determinants of P use efficiency under future CO₂ conditions and suggest new avenues for improving nutrient acquisition in cereal cropping systems.

## Materials and Methods

*Oryza sativa ssp. japonica cv. Nipponbare (wild-type and ospt11-1)* plants were grown in controlled-environment chambers under ambient (400 ppm) or elevated (900 ppm) CO_2_ conditions. Plants were cultivated either in whole-pot experiments across a phosphate supply gradient (by mixing defined amounts of KH_2_PO_4_) or in split-root systems that allowed paired inoculated and noninoculated root compartments within the same plant. *Rhizophagus irregularis* BEG87 was used for mycorrhizal inoculation. Plant biomass, root architecture, phosphate concentration, and content were quantified upon harvest and mycorrhizal colonization determined after trypan blue staining of roots. Dual-radioisotope (^32^P/^33^P) tracing experiments were performed to distinguish symbiotic and direct phosphate uptake pathways. ^32^P was provided to a hyphal compartment not accessible by roots; and ^33^P was provided to the noncolonized root section. Labeled species of P were measured with a liquid scintillation counter. RNA was extracted with a silica column-based method. Root transcriptomes from wild-type and *pt11* plants were analyzed using RNA sequencing (Illumina), differential expression analysis, and gene coexpression analysis. Full experimental details of plant, soils and growth conditions, plant harvests, radiotracer experiments, RNA extraction, qPCR, RNA-seq analyses, and statistical procedures are provided in *SI Appendix, Materials and Methods*.

## Supplementary Material

Appendix 01 (PDF)

Dataset S01 (XLSX)

Dataset S02 (XLSX)

Dataset S03 (XLSX)

Dataset S04 (XLSX)

## Data Availability

RNA-seq data generated in this study have been deposited in the NCBI GSE333245. Processed transcript abundance files (.xprs) for all 21 RNA-seq libraries have been deposited in Figshare (DOI: 10.6084/m9.figshare.32400312).
