# Supplementary material for: Elevated CO2 reinforces PT11-dependent symbiotic phosphate uptake to reprogram root nutrient acquisition in rice
Source: Proc Natl Acad Sci U S A. 2026 Sep 1;123(36):e2606406123. doi: 10.1073/pnas.2606406123 (PMC13552895; doi:10.1073/pnas.2606406123)
Supplement: Supplementary file 1 [file pnas.2606406123.sapp.pdf]

PNAS

Supporting Information for

Elevated CO_2 reinforces symbiotic phosphate uptake to reprogram root nutrient acquisition in rice

Chai Hao Chiu[1*], Mette Grønlund[2*], Thomas Christian de Bang[2*], Stephanie Watts-Williams[2,3,4], Fang Song[5], Krystyna Anna Kelly[5], Iver Jakobsen[2†] and Uta Paszkowski[1†]

[1] Crop Science Centre, Department of Plant Sciences, 93 Lawrence Weaver Road, Cambridge CB3 0LE, United Kingdom

[2] Department of Plant and Environmental Sciences, University of Copenhagen, Thorvaldsensvej 40, DK-1871 Frederiksberg C, Denmark

[3] Centre for Crop Systems Analysis, Wageningen University & Research, 6708 PE, Wageningen, The Netherlands

[4] The Waite Research Institute and The School of Agriculture, Food and Wine, The University of Adelaide, Waite Campus, Urrbrae, South Australia, Australia

[5] Department of Plant Sciences, Downing Street, Cambridge CB2 3EA, United Kingdom

*These authors contributed equally

† Authors for correspondence: Iver Jakobsen (ivja@plen.ku.dk); Uta Paszkowski (up220@cam.ac.uk)

Author Contributions: Conceptualisation, I.J., U.P.; Investigation, M.G., T.C.d.B., S.W-W., S.F., C.H.C..; Methodology, K.K.. Writing: C.H.C. Funding Acquisition, I.J., U.P., T.C.d.B. All authors have contributed to and approved the final manuscript.

Competing Interest Statement: The authors declare no competing interests

Classification: Biological Sciences; Plant Sciences.

Keywords: arbuscular mycorrhiza; symbiosis; rice; elevated CO_2; phosphate

This PDF file contains
SI Materials and Methods
Supplemental Figures S1 to S9
Supplemental Note 1
SI Dataset Legends
Supplemental References

SI Appendix: Materials and Methods

Soil and plants

Oryza sativa ssp *japonica* cultivar Nipponbare (referred to as the wild type hereafter) and mutant *ospt11-1* from (1) were used in this study. Seeds of all genotypes were surface-sterilized (20 min with 1.5% v/v sodium hypochlorite), washed and pre-germinated on 0.8% water-agar plates for 2 days at 25°C. Pre-germinated seedlings were sown (day 0) in small pots containing 160 g of a semi-sterile (15 kGy, 10 MeV electron beam) 1:1 soil/sand mix, which was supplemented with basal nutrients [as per (2)] including 10 mg P kg^{-1} (as KH_2PO_4), and mixed thoroughly. Seedlings grew for 16 d at aCO_2 before being moved to split-pots under respective CO2 regimes [following (3)]. The root system of each plant was divided into two halves of a similar size and planted in a split-root system consisting of two adjoining pots with one root half in each. Pots contained a semi-sterile 1:1 soil/sand mixture, as above, hereafter named "substrate", with 10 mgP kg^{-1} as KH_2PO_4, mixed into the growth medium, resulting in 12±1.3 mgP/kg soil level of 0.5 M $NaHCO_3$-extractable P (1). The split-root treatments for WT plants were (1) NM,M: one root half non-inoculated and the other inoculated with AM fungi; and (ii) NM,NM: both root halves non-inoculated. For mycorrhizal treatments *Rhizophagus irregularis* [previously named *Glomus intraradices (2)*] BEG87 inoculum, consisting of a mixture of dry soil, spores, and root fragments of a *Trifolium subterraneum* pot culture (1), was used. For P radiotracer experiments, five randomly selected pots of each treatment was supplied with the following: in pot (A), 600 g substrate mixed with 262 kBq of carrier-free $H_3{}^{33}PO_4$ P-labelled soil in a small plastic cylinder that was capped with 25 μm nylon mesh at both ends, allowing fungal hyphae, but not roots, to penetrate from the surrounding soil. The HC contained 262 kBq of carrier-free $H_3{}^{32}PO_4$. And in Pot (B), 545 g of unlabeled substrate and 55 g of ^{32}P-labelled soil in a small plastic cylinder that was capped with 25 μm nylon mesh at both ends, allowing fungal hyphae, but not roots, to penetrate from the surrounding soil.The specific activity in 0.5M $NaHCO_3$ extracts of the ^{33}P-labelled soil was 12kBq mg P^{-1}. The specific activity in 0.5M $NaHCO_3$ extracts of the ^{32}P-labelled soil was 162kBq mg P^{-1}.

In the P-response experiment, 2-3 pre-germinated seeds were sown in each pot and thinned to one plant per pot after emergence. Pre-germinated seeds were sown (day 0) in pots containing 1430 g of the substrate described above, but with different P concentrations. Five

soil P concentrations were established by mixing KH_2PO_4 (at 10 mg P kg^{-1}) into the growth medium at 0, 10, 20, 40 and 80 mg P kg^{-1}. These P additions resulted in levels of 0.5 M $NaHCO_3$-extractable P (6) of 4.3 ± 0.3, 7.8 ± 0.1, 11.2± 0.5, 19.8± 0.6 and 39.6 ± 0.9 mg P kg^{-1} soil and are referred to as 0P, 10P, 20P, 40P and 80P hereafter. In Mycorrhizal (M) pots, 100 g of the substrate was substituted with 100 g of *Rhizophagus irregularis* BEG87 inoculum. The inoculum consisted of a mixture of dry soil, spores and root fragments of a *Trifolium subterraneum* pot culture. In the mycorrhizal pots, two layers of unlabelled, non-inoculated soil were weighed into the bottom (200 g) and top (80 g) of each 1430 g pot, to prevent transfer of labelled soil and AM fungal inoculum between pots. NM and M pots received 15 inoculum leaching to all pots, prepared from a suspension of 100 g inoculum l^{-1}. The suspension was sieved through 2 layers of 25μm nylon mesh supported on a 38μm sieve. There were 3 replicates of each treatment, for which all response variables were determined.

In both the split-root and P response experiments, plants were grown in climate chambers with 12/12 h light/dark cycle at 28/20°C. Fluorescent daylight lamps (Osram GmbH, Munich, Germany) provided 500 μmol m^{-2} s^{-1} photosynthetically active radiation (PAR; 400–700 nm). Pots were watered every second day to 60% of water-holding capacity and fertilized with N (as NH_4NO_3) according to need. Plants grown at ambient CO_2, experienced around 450 ppm (chamber set to 385 ppm) and at elevated CO_2, around 900 ppm (chamber set to 1000 ppm). To avoid chamber-specific bias in the experiment, the tables holding the plants were relocated between the two climate chambers every week, together with their corresponding CO_2 treatment.

Harvesting and physiological analyses

Plants were harvested at 49d after sowing for the P gradient experiment, and 37 d after sowing (21d after transplantation to split-pots) for split-root, radiotracer and transcriptome experiments. A subsample (~50 mg) of fresh shoot material was flash-frozen in liquid N_2 and kept at –80 °C. Shoots were dried at 70°C for 48 h and dry weights recorded. Roots were washed, blotted, and weighed, and a weighed subsample for determination of AM colonization was stored in 50% ethanol. Another weighed subsample of root material was flash-frozen in liquid N_2, crushed, and kept at –80 °C until RNA isolation. The remaining root tissue was dried at 70°C for 48 h and dry weights were determined.

Plants used for RNA-seq were harvested over three consecutive days. Because each split-root harvest involved extensive physiological and molecular sampling, all plants could not be harvested at a single time of day. Harvesting commenced approximately 4 h after the onset of the light period each day and continued for 8–9 h. To minimise potential diurnal effects, plants were harvested in 15 balanced groups, each containing one biological replicate of every treatment.

Dried shoot and root samples were oxidized in a 4:1 mixture (v:v) of 65% nitric:70% perchloric acids, and total P was determined by the molybdate blue method using AutoAnalyzer3 (Bran+Luebbe, Norderstedt, Germany). The ^{33}P and ^{32}P in shoot and root tissue was determined in the same digests in a Packard TR 1900 liquid scintillation counter (PerkinElmer, Waltham, MA, USA). Prior to determining the percentage root length colonized by AM fungi, root samples were cleared in 10% KOH and stained with trypan blue following (3).

RNA isolation and real-time qPCR analysis

Total RNA was extracted from ~100 mg of root samples of *O. sativa* wild type and of mutant *ospt11*, using an RNeasy Mini Kit (Qiagen Hilden, Germany) with on-column DNase treatment following the manufacturer's protocol. RNA concentration was measured on a Nanodrop ND-1000 Spectrophotometer (Saveen1 Werner, Malmö, Sweden). cDNA was synthesized from 200 ng of total RNA using dNTP mix (Qiagen) and Expand Reverse Transcriptase (Roche) including Protector RNase inhibitor (Roche). The RNA was further purified using the RNeasy Plant Mini kit (Qiagen Hilden, Germany). The real-time PCR primers (Eurofins MWG operon, Germany) for *OsPT2, OsPT6, OsPT11, OsPT13, Ubiquitin* and *Cyclophilin2,* were described in (1). Gene expression analysis was carried out on five (and four for *pt11* myc roots at eCO_2) replicate plants from each treatment. Real-time PCR analysis was performed using the Rotor Gene 2000 Real Time Cycler (Qiagen). Each 20 µl of PCR contained 8 µl of a 1.25–1 dilution of cDNA, and 12 µl of SYBR Green Master Mix (Fermentas, Thermo Scientific), which included 500 nM of each primer. Samples were heated to 95°C for 10 min, followed by 40 cycles of 15 s at 95°C and 1 min at 60°C. After each PCR, the specificity of the amplification was verified by running a melt curve analysis. Data were normalized to the geometric mean of *Ubiquitin* and *Cyclophilin2*.

RNA-seq library preparation, sequencing, and data analyses

RNA extraction and quality controls were conducted as described above. RNA-seq library was generated with 2 µg of total RNA using TruSeq Stranded mRNA Library Prep Kit High Throughput (Illumina, RS-122-2013) according to the manufacturer's protocol. Library quantity and quality were measured using DNA1000 chip on Bioanalyzer 1200 (Agilent Technology). In total 21 samples **(Fig.3a,b)** were multiplexed and sequenced on NextSeq500 as 2×75 nt paired-end run. Raw fastq reads were cleaned by removing adapter and low-quality sequences using Trimmomatic v0.38 (10). Mapping of cleaned reads was performed by TopHat2 (11) with the default settings against MSU7 version of the rice reference genome [Rice Genome Annotation Project, RGAP, (12)]. The resulting BAM files were subjected to Cufflinks (13) for transcript assembly and then to Cuffquant and Cuffnorm pipeline to quantify the transcript levels. Transcript assembly was guided by the reference MSU7 annotation and all the parameters used in Cufflinks suite were set to the default. To address for outliers in sequencing replicates, non-metric multidimensional scaling was performed. No replicates were discarded. edgeR (14) was used for preprocessing and filtering of RNA-seq data. Differentially expressed genes (DEGs) were identified by using the R package DESeq2 (15) for all selected pairwise comparisons with the threshold of fold-change >1.5 and adjusted p-value < 0.05. DEG heatmaps were plotted by row standardizing the expression values to z-scores. A Z-score shows a single gene expression value relative to the mean of that of all six genotypes.

Principal Component Analysis (PCA)

To examine global sample relationships and major sources of variance, Principal Component Analysis (PCA) was performed on the variance-stabilized transcript counts using the *prcomp* function in R. To minimize stochastic noise, the analysis was restricted to the top 2,000 most variable genes across all samples based on row variance calculations. To optimize the signal-to-noise ratio, row-wise sample variances were calculated using the *sparseMatrixStats* library. Eigenvalues and percentage of explained variance were extracted from the resulting rotation matrix. Ordination plots were generated to project sample scores along the major orthogonal axes (PC1, PC2, and PC3), stratified by experimental condition and inoculation status. Sample clustering patterns were evaluated and visualized by superimposing 95% confidence ellipses for each experimental condition using the *ggforce* framework.(4)

Gene Co-expression Analysis and Clustering Frameworks

To resolve coordinated transcriptional dynamics across experimental conditions, variance-stabilized transformation (VST) expression data were analyzed using complementary clustering strategies. For global landscape characterisation across seven conditions (AWC, AWN, AWM, EWC, EWM, APN, and APM), transcripts were ranked by total row-wise variance across all sample profiles to capture major transcriptomic trends without *a priori* contrast bias. The top 2,000 highly variable genes were selected (**Fig. 3**). For individual replicate profiling, expression values were normalized via row-wise *Z*-scores. For core module definition, VST values were averaged across biological replicates (*n=3* per condition) and subsequently *Z*-score scaled to isolate relative expression trajectories. *K*-means clustering (*k=10*) was executed on the averaged, scaled expression matrix using a fixed random seed (set as 123) to ensure algorithmic reproducibility. Partitioned modules were visualized via heatmaps using *ComplexHeatmap (5)* and dynamically linked to expression trendlines tracking mean cluster trajectories via *circlize* (6). Standard errors of the mean (SEM) were calculated across replicates to monitor intra-module variance.

To dissect variations in arbuscular mycorrhizal (AM) responsiveness under ambient versus elevated CO_2 regimes in wild-type plants (**Fig. 3a–c**), an independent clustering pipeline was applied exclusively to established AM-induced genes. Mean transcript expression levels were calculated for the AWC, AWM, EWC, and EWM treatment groups, followed by $\log_2(x + 1)$ transformation and row-wise *Z*-score standardization. Transcripts exhibiting zero variance were excluded. Agglomerative hierarchical clustering was performed using Ward's minimum variance criterion (`ward.D2` method) on a Euclidean distance matrix to partition the AM-responsive transcriptome into 6 distinct structural modules.

To map transcriptional dependency on *PT11* (**Fig. 4d–f**), target candidate modules exhibiting strong symbiosis-mediated upregulation in wild-type (Modules 3 and 4, of Fig. 3) were selected for subdivision. The mean replicate *Z*-score within the mutant condition (APM) was calculated for each transcript. Transcripts maintaining elevated expression above the global baseline within the mutant profile ($Z>0$) were classified as *PT11*-independent, whereas transcripts dropping below or matching the baseline ($Z\leq0$) were classified as *PT11*-dependent. This approach was designed to capture genes with attenuated but real induction that would not survive a significance threshold applied to the *pt11* dataset alone. The

resulting PT11-dependent and PT11-independent gene sets were inspected and consistent with the purely statistical DEG overlap between AM-induced genes in wild-type and *pt11* roots **(Fig.4d,e)**, supporting the robustness of the classification while providing a richer picture of partial/attenuated responses that a significance-only approach would have discarded.

Variation profiles across the subdivided cohorts were resolved as mean group trajectories±SEM boundaries using *ggplot2*. A selected subset of curated reference loci within these modules was extracted and resolved as an annotated sub-heatmap using *ComplexHeatmap*, where categorical dependency status was mapped as a discrete right-side column annotation block. All analyses were executed in R v4.3.2.

Cross-reference datasets

To obtain genes regulated by PHR transcription factors, we used the dataset from Shi et al. 2022 (8)(570 genes, see **Dataset S3A**). To obtain genes regulated by SMAX1 in rice, we used the RNAseq dataset from Choi et al. 2020 [1078 genes, (7)] from Supplementary Data S5 (up) and S7 (down), giving **Dataset S3B**. These gene sets were used to investigate if canonical AM signalling and/or phosphate starvation signalling promoting symbiosis was activated under elevated CO_2 in uncolonized rice roots ie. in the EWC vs AWC dataset (**Fig. S4d,e,f**). To supplement the rice MSU genome annotation with gene nomenclature from functionally characterised genes, funRiceGenes (9) along with an internal curated gene set was used.

Supplemental References

1. Estimation of Available Phosphorus in Soils by Extraction with Sodium ... - Sterling Robertson
2. A. Schüßler, C. Walker, “7 Evolution of the ‘Plant-Symbiotic’ Fungal Phylum, Glomeromycota” in *Evolution of Fungi and Fungal-Like Organisms*, (Springer Berlin Heidelberg, 2011), pp. 163–185.
3. P. Kormanik, A. McGraw, “Quantification of vesicular-arbuscular mycorrhizae in plant roots.” in *Methods and Principles of Mycorrhizal Research .*, N. Schenck, Ed. (St Paul, MN: American Phytopathological Society, 1982), pp. 37–45.
4. T. L. Pedersen, Accelerating ggplot2 • ggforce. (2024). Available at: https://ggforce.data-imaginist.com/ [Accessed 25 May 2026].
5. Z. Gu, R. Eils, M. Schlesner, Complex heatmaps reveal patterns and correlations in multidimensional genomic data. *Bioinformatics* **32**, 2847–2849 (2016).
6. Z. Gu, L. Gu, R. Eils, M. Schlesner, B. Brors, circlize Implements and enhances circular visualization in R. *Bioinformatics* **30**, 2811–2812 (2014).

7. J. Choi, *et al.*, The negative regulator SMAX1 controls mycorrhizal symbiosis and strigolactone biosynthesis in rice. *Nat. Commun.* **11**, 1–13 (2020).
8. J. Shi, *et al.*, A phosphate starvation response-regulated receptor-like kinase, OsADK1, is required for mycorrhizal symbiosis and phosphate starvation responses. *New Phytologist* **236**, 2282–2293 (2022).
9. F. Huang, *et al.*, New Data and New Features of the FunRiceGenes (Functionally Characterized Rice Genes) Database: 2021 Update. *Rice 2022 15:1* **15**, 23- (2022).

Supplemental Figures

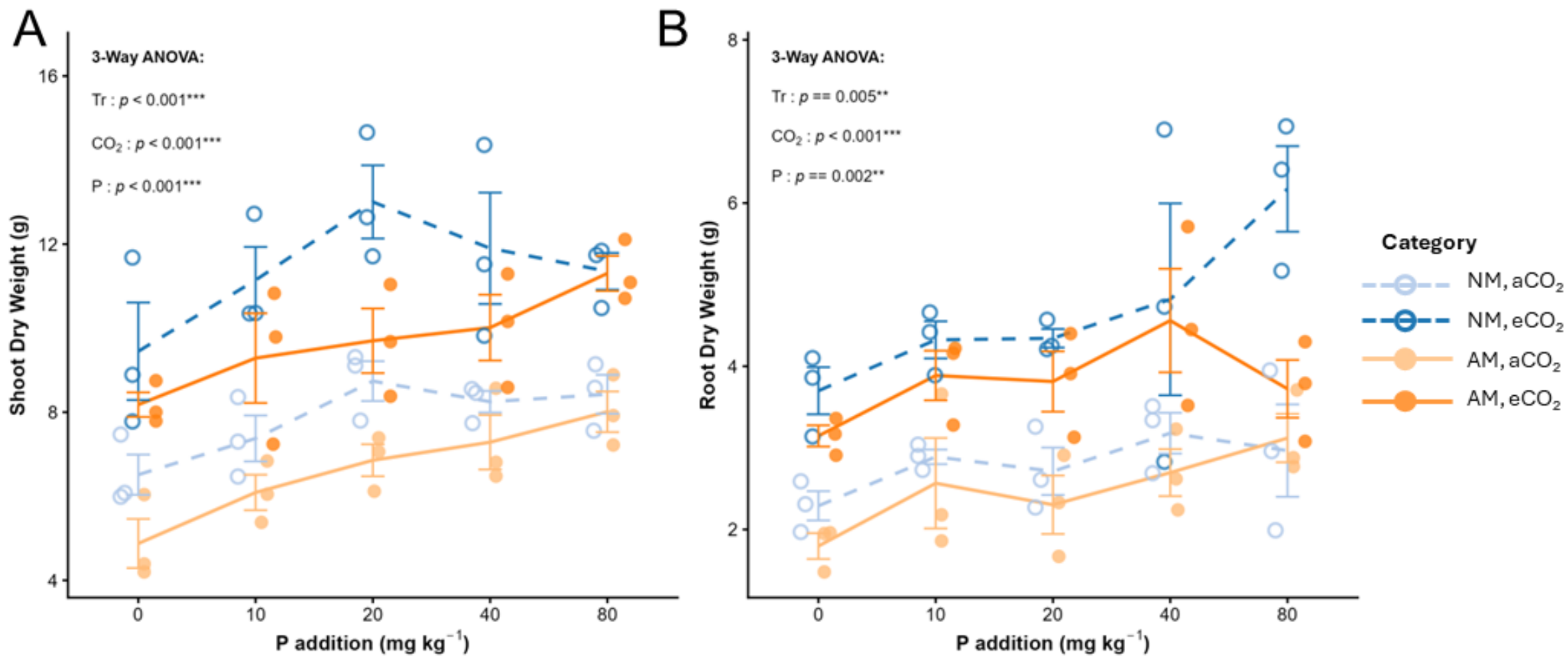

Figure S1: (A) Shoot and (B) root dry weights, in support of Figure 1. Bars represent means ± SEM with data points from individual plants overlaid. Colour scheme consistent with above, blue indicating non-mycorrhizal (NM) plants, orange indicating mycorrhizal (AM) plants, colour intensity representing CO_2 treatment, with elevated CO_2 being darker. Text insets provide 3-way ANOVA *P*-values for AM inoculation (AM), CO_2, and P addition (P).

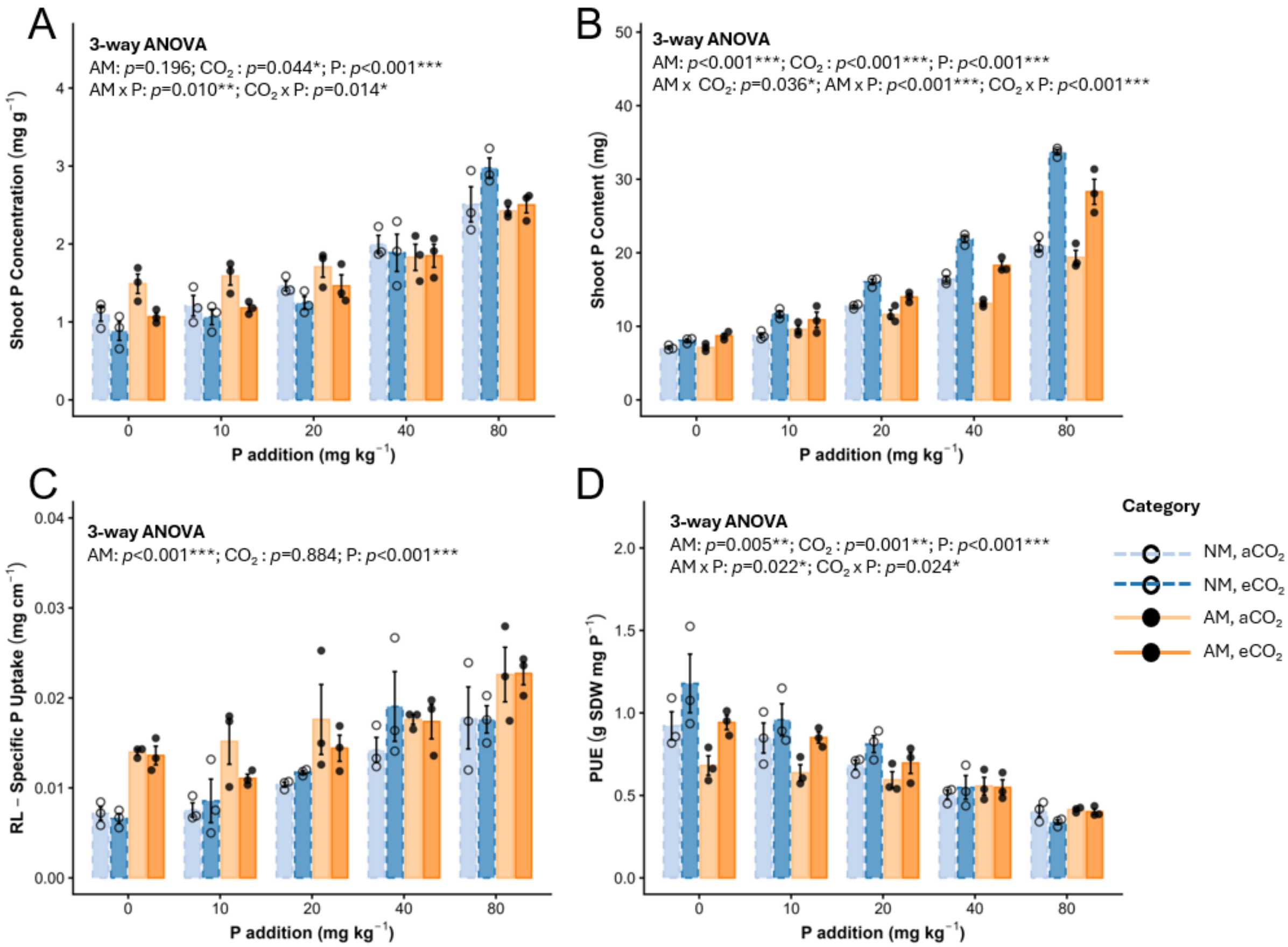

Figure S2: Phosphorus (P) and CO_2 supply increases shoot P accumulation and biomass, supporting Figure 1. (A) Shoot P concentrations, **(B)** shoot P content, **(C)** root length specific P uptake (mg of P per g of root dry weight) and **(D)** P use efficiency (PUE) measured across increasing P levels, calculated as g of shoot dry weight (SDW) per mg of P. Bars indicate means ± SE; points represent individual biological replicates. Colour scheme consistent with above, blue indicating non-mycorrhizal (NM) plants, orange indicating mycorrhizal (AM) plants, colour intensity representing CO_2 treatment, with elevated CO_2 being darker. Text insets provide 3-way ANOVA *P*-values for AM inoculation (AM), CO_2, and P addition (P).

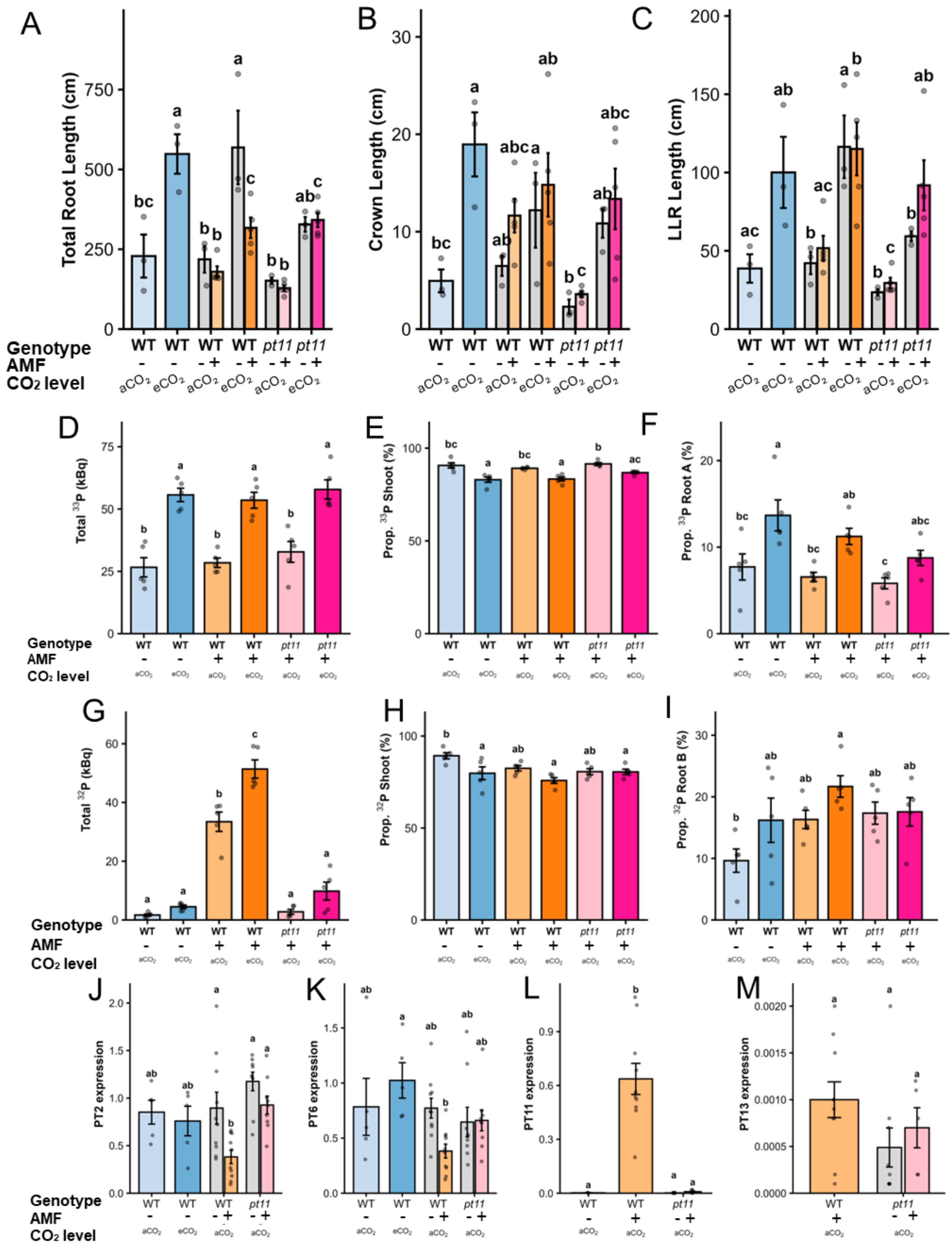

Figure S3: Impact of mycorrhizal colonisation and elevated CO_2 on root architecture, P uptake and phosphate transporter expression in a split-root system, supporting Figure 2. (A) Total root length (cm), **(B)** Crown and large lateral root (LLR) length (cm), **(D)** Proportion of fine lateral roots (%). Data are shown for wild-type (WT) and *pt11* mutant plants grown under ambient (a) and elevated (e) CO_2 conditions. Tracing the direct P uptake pathway using ^{33}P, with **(D)** Total amount in plant, **(E)** Proportion (%) in shoots, and **(F)** Proportion in Root A, where ^{33}P was provided. Tracing the symbiotic uptake pathway using ^{32}P, with **(G)** Total amount in plant, **(H)** Proportion in shoot and **(I)** Proportion in Root B, where AM fungi and 32P were provided. Relative expression of **(J)** *PT2*, **(K)** *PT6*, **(L)** *PT11*, and **(M)** *PT13* in split-root systems of wild type (WT) and *pt11* plants under ambient (aCO_2) or elevated CO_2 (eCO_2) conditions, with or without symbiosis (+ or -, respectively) in the respective compartments. Bars represent means ± SE, and points indicate individual biological replicates. Root compartments (RA and RB) are shown as grouped bars within each treatment Different letters above bars indicate significant differences among treatments within each panel based on one-way ANOVA followed by Tukey's HSD post hoc test ($P < 0.05$). *AMF* denotes inoculation status: "**–**" denotes mock-inoculated control plants (both sides being non-mycorrhizal, NM), and split barplots denotes split-root plants where one root half was inoculated with *Rhizophagus irregularis* (+)*,* and the other half uninoculated (-). For each treatment, the bars are paired to represent the two halves of the same root system: Grey bars represent the uncolonized root half (Root A). In split-root plants, this is the systemic, non-inoculated side. Colors distinguish treatment groups: Blue = Mock-inoculated; Orange = WT mycorrhizal-inoculated; Pink = *pt11* mutant, mycorrhizal-inoculated. Bars represent mean SE (n=4-7). Different letters above the bars indicate statistically significant differences between all root halves across all treatments, determined by one-way ANOVA followed by Tukey's HSD post-hoc test ($p < 0.05$).

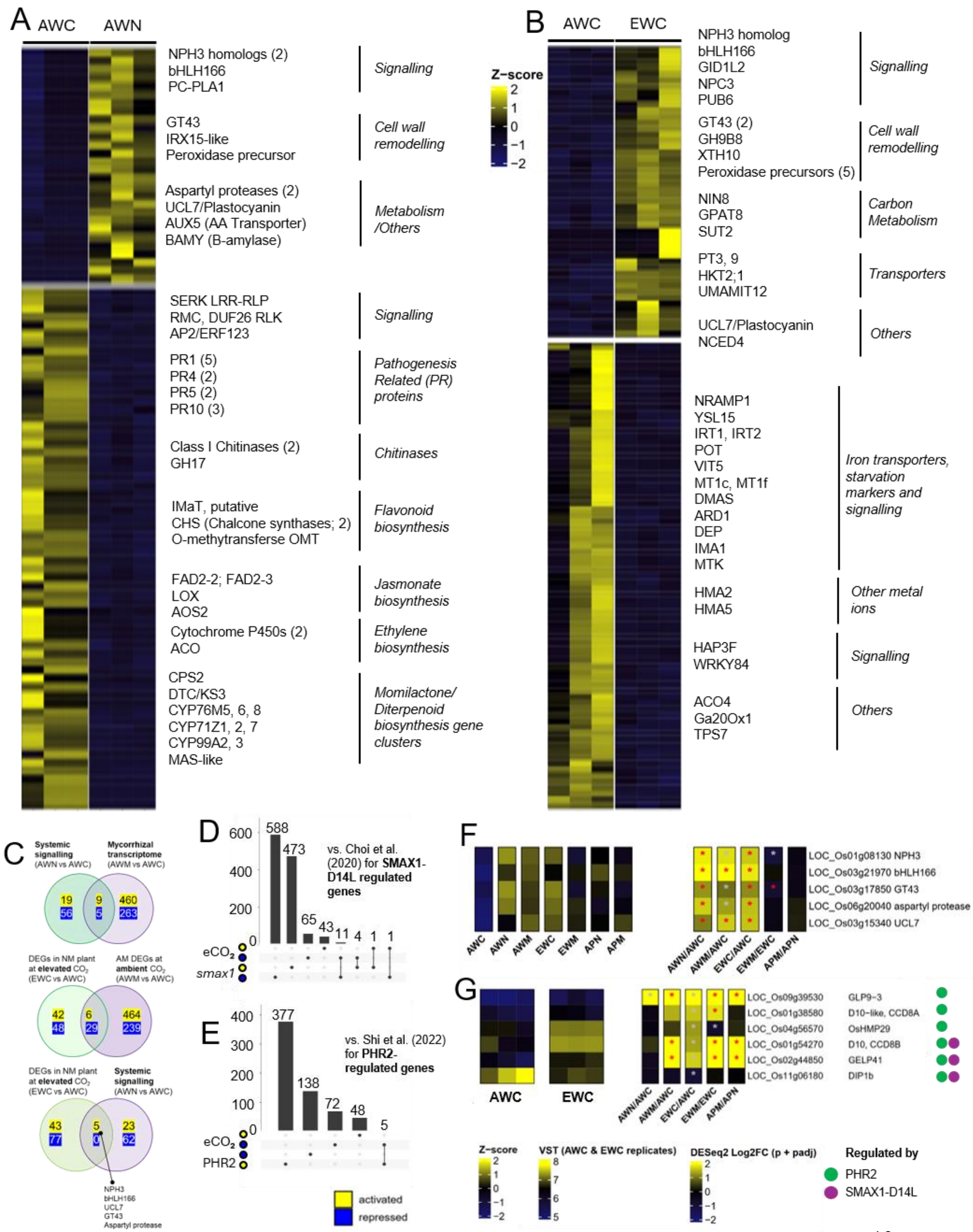

A AWC AWN
NPH3 homologs (2)
bHLH166
PC-PLA1
Signalling
GT43
IRX15-like
Peroxidase precursor
Cell wall remodelling
Aspartyl proteases (2)
UCL7/Plastocyanin
AUX5 (AA Transporter)
BAMY (B-amylase)
Metabolism /Others
SERK LRR-RLP
RMC, DUF26 RLK
AP2/ERF123
Signalling
PR1 (5)
PR4 (2)
PR5 (2)
PR10 (3)
Pathogenesis Related (PR) proteins
Class I Chitinases (2)
GH17
Chitinases
IMaT, putative
CHS (Chalcone synthases; 2)
O-methytransferse OMT
Flavonoid biosynthesis
FAD2-2; FAD2-3
LOX
AOS2
Jasmonate biosynthesis
Cytochrome P450s (2)
ACO
Ethylene biosynthesis
CPS2
DTC/KS3
CYP76M5, 6, 8
CYP71Z1, 2, 7
CYP99A2, 3
MAS-like
Momilactone/ Diterpenoid biosynthesis gene clusters
Z-score
2 1 0 −1 −2
B AWC EWC
NPH3 homolog
bHLH166
GID1L2
NPC3
PUB6
Signalling
GT43 (2)
GH9B8
XTH10
Peroxidase precursors (5)
Cell wall remodelling
NIN8
GPAT8
SUT2
Carbon Metabolism
PT3, 9
HKT2;1
UMAMIT12
Transporters
UCL7/Plastocyanin
NCED4
Others
NRAMP1
YSL15
IRT1, IRT2
POT
VIT5
MT1c, MT1f
DMAS
ARD1
DEP
IMA1
MTK
Iron transporters, starvation markers and signalling
HMA2
HMA5
Other metal ions
HAP3F
WRKY84
Signalling
ACO4
Ga20Ox1
TPS7
Others
C
Systemic signalling (AWN vs AWC)
Mycorrhizal transcriptome (AWM vs AWC)
19 56 9 5 460 263
DEGs in NM plant at elevated CO2 (EWC vs AWC)
AM DEGs at ambient CO2 (AWM vs AWC)
42 48 6 29 464 239
DEGs in NM plant at elevated CO2 (EWC vs AWC)
Systemic signalling (AWN vs AWC)
43 77 5 0 23 62
NPH3
bHLH166
UCL7
GT43
Aspartyl protease
D
600 400 200 0
588 473 65 43 11 4 1 1
vs. Choi et al. (2020) for SMAX1-D14L regulated genes
eCO2
smax1
E
400 300 200 100 0
377 138 72 48 5
vs. Shi et al. (2022) for PHR2-regulated genes
eCO2
PHR2
activated
repressed
F
AWC AWN AWM EWC EWM APN APM
AWN/AWC AWM/AWC EWC/AWC EWM/EWC APM/APN
LOC_Os01g08130 NPH3
LOC_Os03g21970 bHLH166
LOC_Os03g17850 GT43
LOC_Os06g20040 aspartyl protease
LOC_Os03g15340 UCL7
G
AWC EWC
AWN/AWC AWM/AWC EWC/AWC EWM/EWC APM/APN
LOC_Os09g39530 GLP9-3
LOC_Os01g38580 D10-like, CCD8A
LOC_Os04g56570 OsHMP29
LOC_Os01g54270 D10, CCD8B
LOC_Os02g44850 GELP41
LOC_Os11g06180 DIP1b
Z-score
2 1 0 −1 −2
VST (AWC & EWC replicates)
8 7 6 5
DESeq2 Log2FC (p + padj)
2 1 0 −1 −2
Regulated by
PHR2
SMAX1-D14L

Figure S4. Systemic mycorrhizal signalling and elevated CO_2–responsive gene expression in roots. (A, B) Heatmaps of z-score–normalized expression for differentially expressed genes (DEGs) identified by the contrasts AWC vs. AWN (systemic transcriptional responses in non-colonized half of split-root plants, **A**) and EWC vs. AWC (genes regulated by elevated CO_2 in non-mycorrhizal roots, **B**) Notable genes, classified according to their functions, are highlighted. See **Figure S4, S5** for expanded gene list with gene identifiers. Rows represent individual genes and columns represent Z-scores of individual replicates. **(C)** Venn diagrams showing the overlap of up-regulated (yellow) and down-regulated (blue) DEGs between **(C)** systemic mycorrhizal signalling (AWC vs. AWN) and mycorrhizal transcriptome (AWM vs. AWC); elevated CO_2 responses (EWC vs. AWC) with mycorrhizal transcriptome and with systemic signalling. **(D,E)** Transcriptome of elevated CO_2 responses were also compared against published datasets of genes known to be regulated by SMAX1-D3-D14L **(D)** and genes known to be regulated by PHR2 **(E)**. **(F)** Genes defining the core overlap between systemic response and elevated CO_2 response, see **Figure S4** for full list. The left block of heatmap displays the Z-score–scaled variance-stabilized (VST) expression values based on group means for each condition (AWC, AWN, AWM, EWC, EWM, APN, APM) in (in **F**; or individual replicates, in **G**). Z-scores were calculated per gene across conditions, where yellow indicates higher-than-average expression and blue indicates lower-than-average expression. Right block displays the log2FC across the seven key comparisons in this study. Differential expression was defined using false discovery rate–adjusted $P < 0.05$. **red** stars indicate adjusted p-value (padj) < 0.05, while **grey** stars indicate p-value < 0.05 but padj ≥ 0.05. Gene identifiers and functional annotations shown on the right.

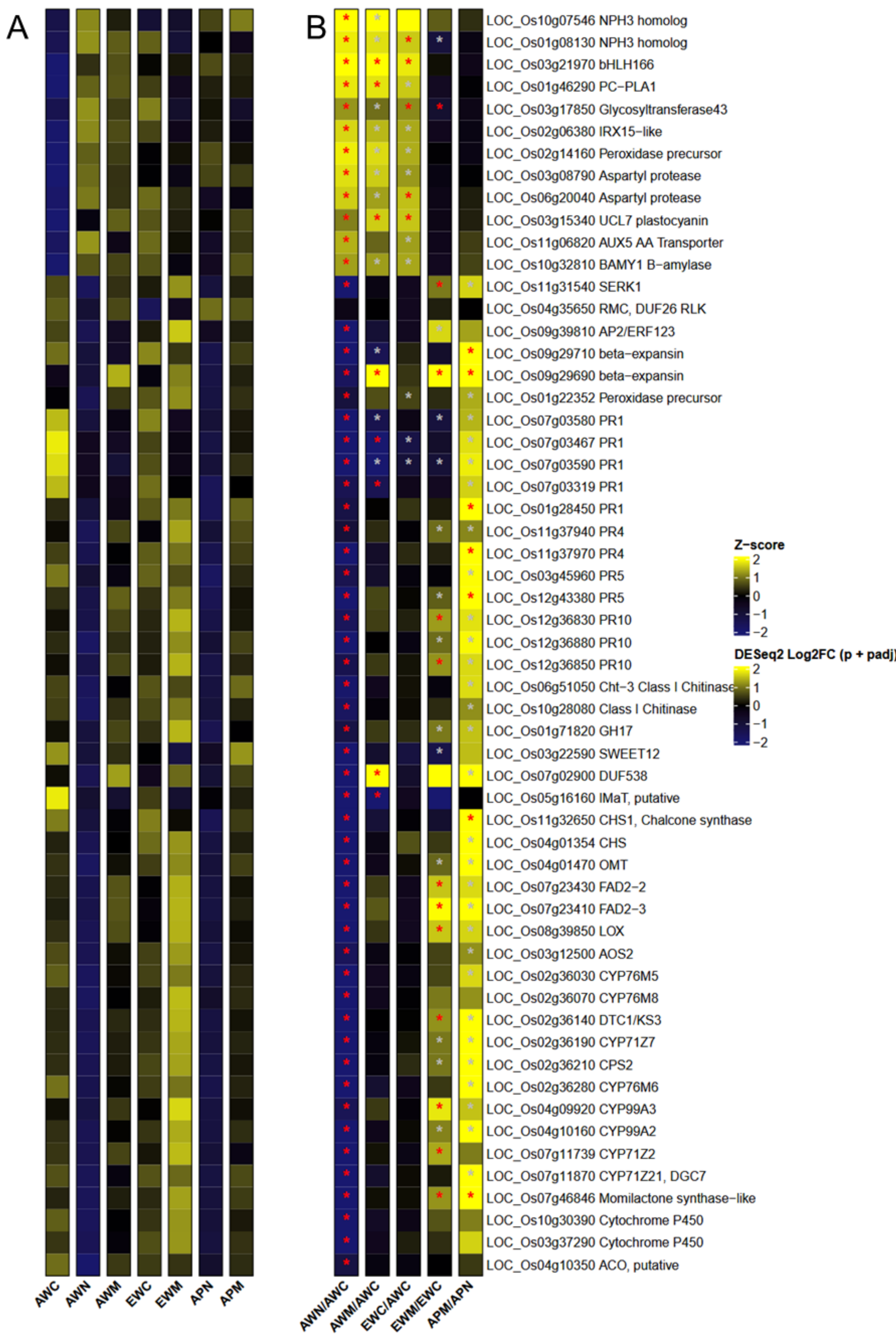
A
B
LOC_Os10g07546 NPH3 homolog
LOC_Os01g08130 NPH3 homolog
LOC_Os03g21970 bHLH166
LOC_Os01g46290 PC−PLA1
LOC_Os03g17850 Glycosyltransferase43
LOC_Os02g06380 IRX15−like
LOC_Os02g14160 Peroxidase precursor
LOC_Os03g08790 Aspartyl protease
LOC_Os06g20040 Aspartyl protease
LOC_Os03g15340 UCL7 plastocyanin
LOC_Os11g06820 AUX5 AA Transporter
LOC_Os10g32810 BAMY1 B−amylase
LOC_Os11g31540 SERK1
LOC_Os04g35650 RMC, DUF26 RLK
LOC_Os09g39810 AP2/ERF123
LOC_Os09g29710 beta−expansin
LOC_Os09g29690 beta−expansin
LOC_Os01g22352 Peroxidase precursor
LOC_Os07g03580 PR1
LOC_Os07g03467 PR1
LOC_Os07g03590 PR1
LOC_Os07g03319 PR1
LOC_Os01g28450 PR1
LOC_Os11g37940 PR4
LOC_Os11g37970 PR4
LOC_Os03g45960 PR5
LOC_Os12g43380 PR5
LOC_Os12g36830 PR10
LOC_Os12g36880 PR10
LOC_Os12g36850 PR10
LOC_Os06g51050 Cht−3 Class I Chitinase
LOC_Os10g28080 Class I Chitinase
LOC_Os01g71820 GH17
LOC_Os03g22590 SWEET12
LOC_Os07g02900 DUF538
LOC_Os05g16160 IMaT, putative
LOC_Os11g32650 CHS1, Chalcone synthase
LOC_Os04g01354 CHS
LOC_Os04g01470 OMT
LOC_Os07g23430 FAD2−2
LOC_Os07g23410 FAD2−3
LOC_Os08g39850 LOX
LOC_Os03g12500 AOS2
LOC_Os02g36030 CYP76M5
LOC_Os02g36070 CYP76M8
LOC_Os02g36140 DTC1/KS3
LOC_Os02g36190 CYP71Z7
LOC_Os02g36210 CPS2
LOC_Os02g36280 CYP76M6
LOC_Os04g09920 CYP99A3
LOC_Os04g10160 CYP99A2
LOC_Os07g11739 CYP71Z2
LOC_Os07g11870 CYP71Z21, DGC7
LOC_Os07g46846 Momilactone synthase−like
LOC_Os10g30390 Cytochrome P450
LOC_Os03g37290 Cytochrome P450
LOC_Os04g10350 ACO, putative
Z−score
2
1
0
−1
−2
DESeq2 Log2FC (p + padj)
2
1
0
−1
−2
AWC
AWN
AWM
EWC
EWM
APN
APM
AWN/AWC
AWM/AWC
EWC/AWC
EWM/EWC
APM/APN

Figure S5: DEGs of the systemic response. Transcriptome of the uncolonized half of the split root is compared against a completely naïve, uninoculated plant, supporting Figure S4A. (A) Z-score–scaled variance-stabilized (VST) expression values based on group means for each condition (AWC, AWN, AWM, EWC, EWM, APN, APM). Z-scores were calculated per gene across conditions, where yellow indicates higher-than-average expression and blue indicates lower-than-average expression. **(B)** log2FC across the seven key comparisons in this study. Differential expression was defined using false discovery rate–adjusted $P < 0.05$. **red** stars indicate adjusted p-value (padj) < 0.05, while **grey** stars indicate p-value < 0.05 but padj ≥ 0.05. Gene identifiers and functional annotations are shown on the right.

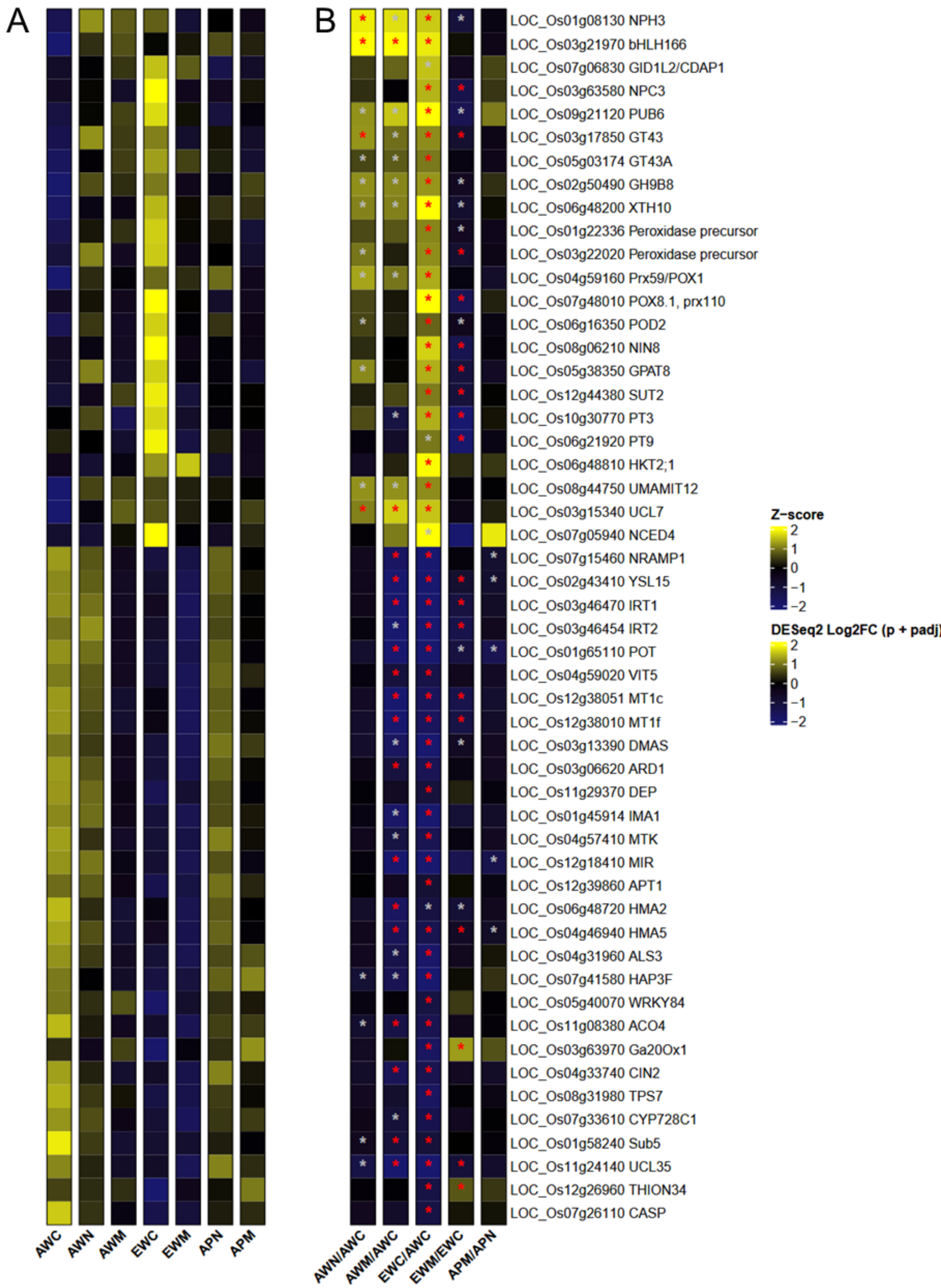

Figure S6: Genes underpinning the elevated CO_2 response in the asymbiotic context. Transcriptomes of completely uninoculated plants at ambient and elevated CO_2 were compared, supporting Figure S4B. (A) Z-score–scaled variance-stabilized (VST) expression values based on group means for each condition (AWC, AWN, AWM,

EWC, EWM, APN, APM). Z-scores were calculated per gene across conditions, where yellow indicates higher-than-average expression and blue indicates lower-than-average expression. **(B)** log2FC across the seven key comparisons in this study. Annotated genes of significant biological functions shown here. Differential expression was defined using false discovery rate–adjusted *P* < 0.05. **red** stars indicate adjusted p-value (padj) < 0.05, while **grey** stars indicate p-value < 0.05 but padj ≥ 0.05. Gene identifiers and functional annotations are shown on the right.

Supplemental Note 1: Detailed Description of Genes in Modules 1 to 6 in Figure 5. Supporting Figure 5 and Figure S6.

Module 1 comprised genes strongly induced by AM symbiosis under ambient CO_2 but showing reduced induction under elevated CO_2 due to elevated baseline expression in non-colonised roots, consistent with a priming effect of elevated CO_2. This module included *bHLH166, UCL7* and also contained defence-related genes and putative jasmonate signalling components (thionins), suggesting that elevated CO_2 may reshape basal defence and biotic interaction pathways in a manner that may facilitate colonisation **(Fig. 4b,c; Fig. S7)**. Although many genes in this module have not been functionally characterised in the context of AM symbiosis, they represent candidate regulators of enhanced colonisation under elevated CO_2; among these, *RAI1* has been implicated in immunity signalling and *CNGC14* in promoting plant stress tolerance.

Module 2 consisted of genes robustly induced by AM symbiosis irrespective of CO_2 concentration and represents a core symbiotic transcriptional program. This module includes canonical AM marker and symbiosis-associated genes required for signalling and bidirectional nutrient exchange at arbuscules, including *AM1*, *AM3*, *ARK1/AM14*, *RAM1*, *FatM, RAM2*, *STR2*, *PT11, PT13*, and *NPF4.5* amongst many others **(Fig. 4b,c; Fig. S7)**. Importantly, these genes were not induced in non-colonised root compartments of the same plants (AWN vs AWC), and many have previously been characterised through promoter–reporter analyses in rice and other species ((29, 47–54)). These observations further support that activation of the core AM transcriptional program is locally restricted to colonised roots and is robustly captured by this experimental system.

Module 3 comprised genes weakly induced by AM symbiosis under ambient CO_2 but strongly induced under elevated CO_2, making this module particularly informative for understanding CO_2-dependent modulation of symbiotic processes. This module was represented by enzymes involved in specialised metabolism, including numerous cytochrome P450s, jasmonate biosynthesis enzymes, and defence-associated genes such as *PR10* family members **(Fig. 4b,c; Fig. S7)**. Notably, multiple components of the mevalonate (MVA) and MEP pathways (*DXS, DXR, IspF, IspG, IspH*) and genes involved in momilactone biosynthesis *(DTC, DTC2, CYP76M17, CYP71Z2)* were strongly upregulated. Momilactones are

diterpenoid phytoalexins typically associated with chemical defence in rice (37), and their pronounced induction specifically in mycorrhizal roots under elevated CO_2 suggests a heightened activation of defence-related secondary metabolism during symbiosis. The spatial and temporal dynamics of this response within colonised roots remain unresolved; however, these data raise the possibility that regulated defence activation may contribute to symbiotic control, potentially linked to later stages of arbuscule turnover or containment.

Module 4 was characterised by broad repression of nutrient transport and associated regulatory pathways during symbiosis. This module included downregulation of direct phosphate uptake transporters (*PT2*) and purple acid phosphatases, as well as extensive suppression of iron transporters, silicon transporters (*Lsi1*, *Lsi2*, *Lsi3*), potassium transporters, and multiple *WRKY* transcription factors. Most strikingly, genes involved in Casparian strip formation, including *MYB36b* and its downstream targets *CASP1* and *CASP2* **(Fig. 4b,c; Fig. S7)**, were repressed (55, 56), suggestive of the remodelling of endodermal diffusion barriers during symbiosis. While the precise root zones in which this repression occurs remain unknown, these changes suggest altered apoplastic connectivity and selective barrier properties that may influence nutrient fluxes, fungal ingress, and containment within colonised roots.

Modules 5 and 6 revealed that mycorrhizal roots under elevated CO_2 exhibit an even stronger suppression of direct phosphate uptake and nutrient starvation responses. Multiple *PHT1* family members (*PT6*, *PT7*, *PT10*, *PT3*, *PT8b*) were downregulated, accompanied by reduced expression of *SPX1* and classical phosphate starvation markers (*IPS1*, *MGD3*, *GDPD5*). In addition, several nitrogen transport and assimilation genes (*NRT1.1a*, *NRT2.3*, *AMT1;2*, *DUR3*) were suppressed, indicating coordinated attenuation of direct nutrient acquisition pathways **(Fig. 4b,c; Fig. S7)**. Notably, this module also included *SUNN-a/FON1*, an ortholog of CLAVATA-clade receptor-like kinases implicated in negative autoregulation of mycorrhizal colonisation in shoots of legumes and *Brachypodium distachyon (57–59)*. Although its role in roots remains unclear, reduced expression of this pathway under elevated CO_2 may contribute to increased colonisation and reduced sensitivity to phosphate-mediated suppression.

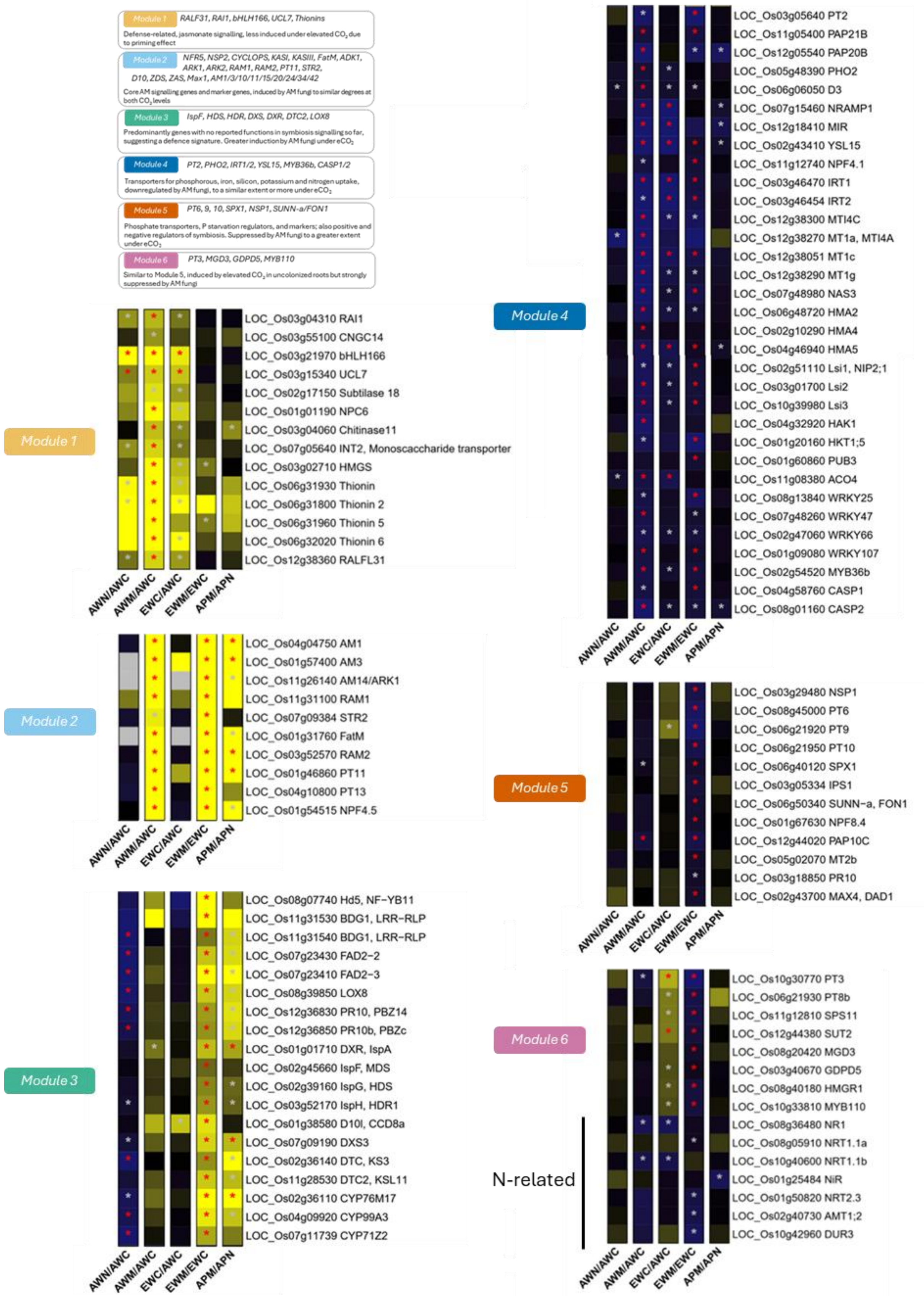

Module 1
RALF31, RAI1, bHLH166, UCL7, Thionins
Defense-related, jasmonate signalling, less induced under elevated CO2 due to priming effect
Module 2
NFR5, NSP2, CYCLOPS, KASI, KASIII, FatM, ADK1, ARK1, ARK2, RAM1, RAM2, PT11, STR2, D10, ZDS, ZAS, Max1, AM1/3/10/11/15/20/24/34/42
Core AM signalling genes and marker genes, induced by AM fungi to similar degrees at both CO2 levels
Module 3
IspF, HDS, HDR, DXS, DXR, DTC2, LOX8
Predominantly genes with no reported functions in symbiosis signalling so far, suggesting a defence signature. Greater induction by AM fungi under eCO2
Module 4
PT2, PHO2, IRT1/2, YSL15, MYB36b, CASP1/2
Transporters for phosphorous, iron, silicon, potassium and nitrogen uptake, downregulated by AM fungi, to a similar extent or more under eCO2
Module 5
PT6, 9, 10, SPX1, NSP1, SUNN-a/FON1
Phosphate transporters, P starvation regulators, and markers; also positive and negative regulators of symbiosis. Suppressed by AM fungi to a greater extent under eCO2
Module 6
PT3, MGD3, GDPD5, MYB110
Similar to Module 5, induced by elevated CO2 in uncolonized roots but strongly suppressed by AM fungi
Module 1
LOC_Os03g04310 RAI1
LOC_Os03g55100 CNGC14
LOC_Os03g21970 bHLH166
LOC_Os03g15340 UCL7
LOC_Os02g17150 Subtilase 18
LOC_Os01g01190 NPC6
LOC_Os03g04060 Chitinase11
LOC_Os07g05640 INT2, Monoscaccharide transporter
LOC_Os03g02710 HMGS
LOC_Os06g31930 Thionin
LOC_Os06g31800 Thionin 2
LOC_Os06g31960 Thionin 5
LOC_Os06g32020 Thionin 6
LOC_Os12g38360 RALFL31
AWN/AWC
AWM/AWC
EWC/AWC
EWM/EWC
APM/APN
Module 2
LOC_Os04g04750 AM1
LOC_Os01g57400 AM3
LOC_Os11g26140 AM14/ARK1
LOC_Os11g31100 RAM1
LOC_Os07g09384 STR2
LOC_Os01g31760 FatM
LOC_Os03g52570 RAM2
LOC_Os01g46860 PT11
LOC_Os04g10800 PT13
LOC_Os01g54515 NPF4.5
AWN/AWC
AWM/AWC
EWC/AWC
EWM/EWC
APM/APN
Module 3
LOC_Os08g07740 Hd5, NF-YB11
LOC_Os11g31530 BDG1, LRR-RLP
LOC_Os11g31540 BDG1, LRR-RLP
LOC_Os07g23430 FAD2-2
LOC_Os07g23410 FAD2-3
LOC_Os08g39850 LOX8
LOC_Os12g36830 PR10, PBZ14
LOC_Os12g36850 PR10b, PBZc
LOC_Os01g01710 DXR, IspA
LOC_Os02g45660 IspF, MDS
LOC_Os02g39160 IspG, HDS
LOC_Os03g52170 IspH, HDR1
LOC_Os01g38580 D10l, CCD8a
LOC_Os07g09190 DXS3
LOC_Os02g36140 DTC, KS3
LOC_Os11g28530 DTC2, KSL11
LOC_Os02g36110 CYP76M17
LOC_Os04g09920 CYP99A3
LOC_Os07g11739 CYP71Z2
AWN/AWC
AWM/AWC
EWC/AWC
EWM/EWC
APM/APN
Module 4
LOC_Os03g05640 PT2
LOC_Os11g05400 PAP21B
LOC_Os12g05540 PAP20B
LOC_Os05g48390 PHO2
LOC_Os06g06050 D3
LOC_Os07g15460 NRAMP1
LOC_Os12g18410 MIR
LOC_Os02g43410 YSL15
LOC_Os11g12740 NPF4.1
LOC_Os03g46470 IRT1
LOC_Os03g46454 IRT2
LOC_Os12g38300 MTI4C
LOC_Os12g38270 MT1a, MTI4A
LOC_Os12g38051 MT1c
LOC_Os12g38290 MT1g
LOC_Os07g48980 NAS3
LOC_Os06g48720 HMA2
LOC_Os02g10290 HMA4
LOC_Os04g46940 HMA5
LOC_Os02g51110 Lsi1, NIP2;1
LOC_Os03g01700 Lsi2
LOC_Os10g39980 Lsi3
LOC_Os04g32920 HAK1
LOC_Os01g20160 HKT1;5
LOC_Os01g60860 PUB3
LOC_Os11g08380 ACO4
LOC_Os08g13840 WRKY25
LOC_Os07g48260 WRKY47
LOC_Os02g47060 WRKY66
LOC_Os01g09080 WRKY107
LOC_Os02g54520 MYB36b
LOC_Os04g58760 CASP1
LOC_Os08g01160 CASP2
AWN/AWC
AWM/AWC
EWC/AWC
EWM/EWC
APM/APN
Module 5
LOC_Os03g29480 NSP1
LOC_Os08g45000 PT6
LOC_Os06g21920 PT9
LOC_Os06g21950 PT10
LOC_Os06g40120 SPX1
LOC_Os03g05334 IPS1
LOC_Os06g50340 SUNN-a, FON1
LOC_Os01g67630 NPF8.4
LOC_Os12g44020 PAP10C
LOC_Os05g02070 MT2b
LOC_Os03g18850 PR10
LOC_Os02g43700 MAX4, DAD1
AWN/AWC
AWM/AWC
EWC/AWC
EWM/EWC
APM/APN
Module 6
LOC_Os10g30770 PT3
LOC_Os06g21930 PT8b
LOC_Os11g12810 SPS11
LOC_Os12g44380 SUT2
LOC_Os08g20420 MGD3
LOC_Os03g40670 GDPD5
LOC_Os08g40180 HMGR1
LOC_Os10g33810 MYB110
LOC_Os08g36480 NR1
LOC_Os08g05910 NRT1.1a
LOC_Os10g40600 NRT1.1b
LOC_Os01g25484 NiR
LOC_Os01g50820 NRT2.3
LOC_Os02g40730 AMT1;2
LOC_Os10g42960 DUR3
N-related
AWN/AWC
AWM/AWC
EWC/AWC
EWM/EWC
APM/APN

Figure S7: Selected genes that highlight the membership of the respective modules 1-6 in Figure 4. All heatmap blocks display log2FC across the seven key comparisons in this study. Differential expression was defined using false discovery rate–adjusted $P < 0.05$. **red** stars indicate adjusted p-value (padj) < 0.05, while **grey** stars indicate p-value < 0.05 but padj ≥ 0.05. Gene identifiers and functional annotations are shown on the right. Grey cells indicate missing values (NA), resulting from insufficient expression or lack of variance for a given gene in the corresponding condition or contrast.

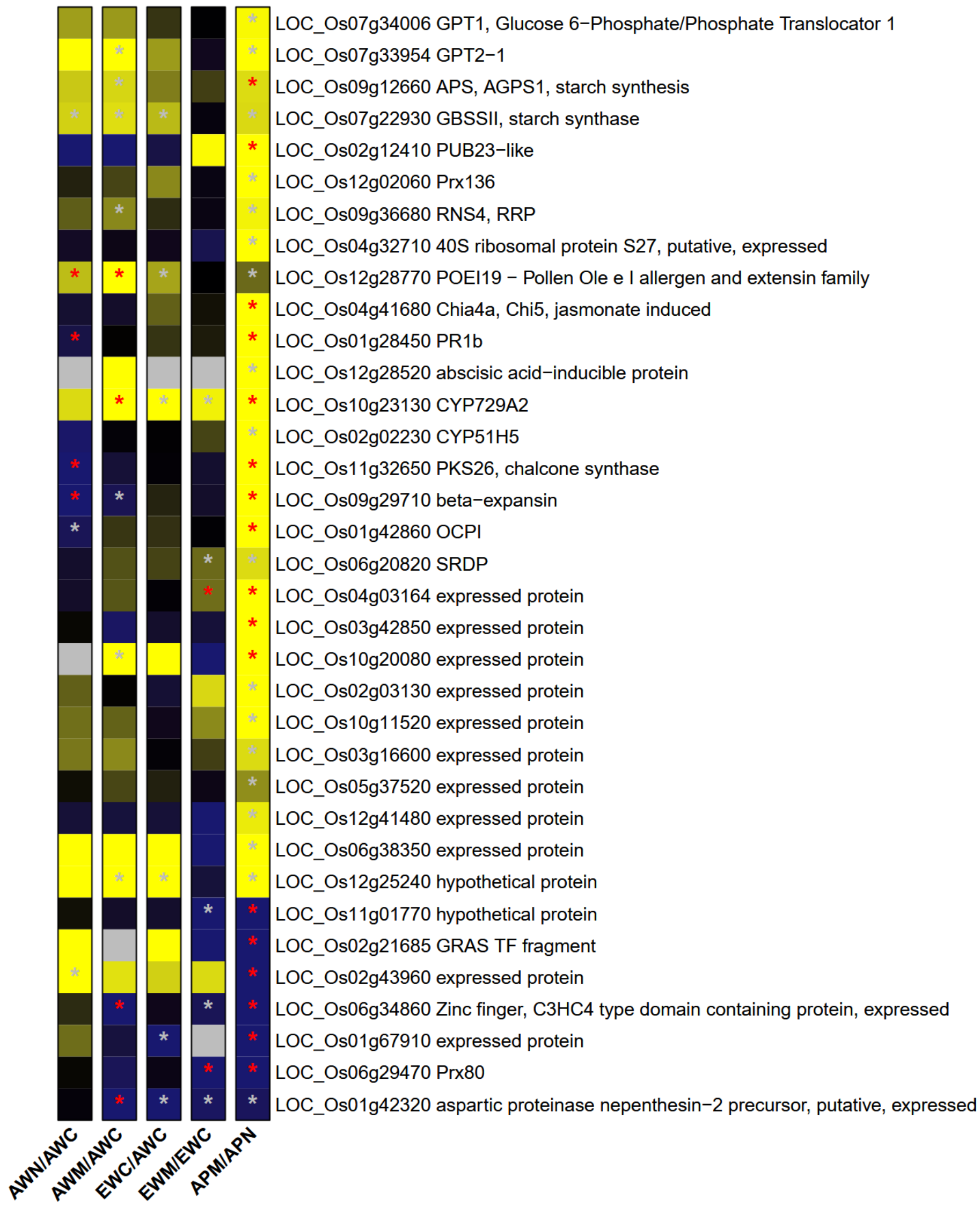

Figure S8: Genes misregulated in *pt11* mutants, supporting Figure 5. All heatmap blocks display log2FC across the seven key comparisons in this study. Differential expression was defined using false discovery rate–adjusted $P < 0.05$. **red** stars indicate adjusted p-value (padj) < 0.05, while **grey** stars indicate p-value < 0.05 but padj ≥ 0.05. Gene identifiers and functional annotations are shown on the right. Grey cells indicate missing values (NA), resulting from insufficient expression or lack of variance for a given gene in the corresponding condition or contrast.

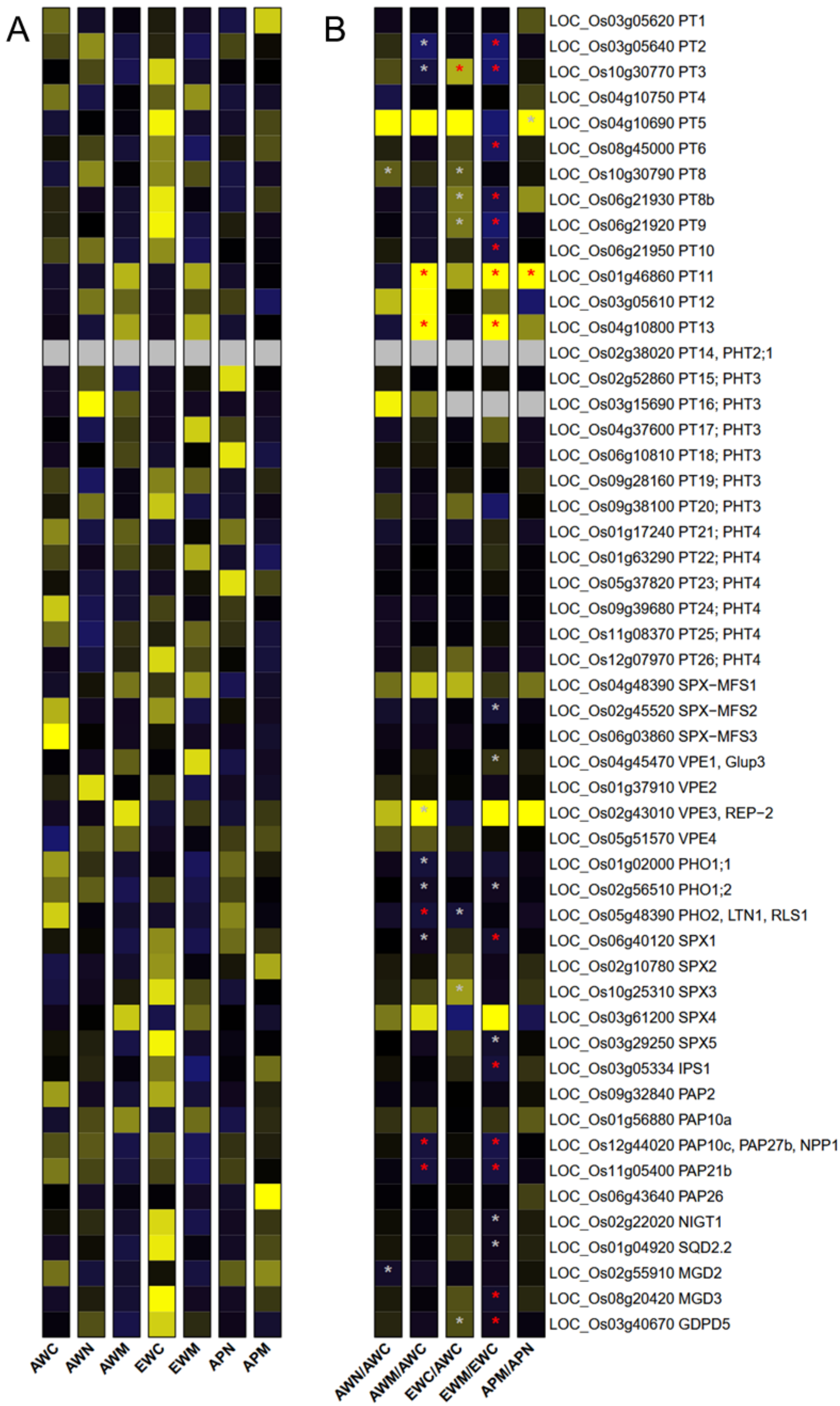

A
B
LOC_Os03g05620 PT1
LOC_Os03g05640 PT2
LOC_Os10g30770 PT3
LOC_Os04g10750 PT4
LOC_Os04g10690 PT5
LOC_Os08g45000 PT6
LOC_Os10g30790 PT8
LOC_Os06g21930 PT8b
LOC_Os06g21920 PT9
LOC_Os06g21950 PT10
LOC_Os01g46860 PT11
LOC_Os03g05610 PT12
LOC_Os04g10800 PT13
LOC_Os02g38020 PT14, PHT2;1
LOC_Os02g52860 PT15; PHT3
LOC_Os03g15690 PT16; PHT3
LOC_Os04g37600 PT17; PHT3
LOC_Os06g10810 PT18; PHT3
LOC_Os09g28160 PT19; PHT3
LOC_Os09g38100 PT20; PHT3
LOC_Os01g17240 PT21; PHT4
LOC_Os01g63290 PT22; PHT4
LOC_Os05g37820 PT23; PHT4
LOC_Os09g39680 PT24; PHT4
LOC_Os11g08370 PT25; PHT4
LOC_Os12g07970 PT26; PHT4
LOC_Os04g48390 SPX-MFS1
LOC_Os02g45520 SPX-MFS2
LOC_Os06g03860 SPX-MFS3
LOC_Os04g45470 VPE1, Glup3
LOC_Os01g37910 VPE2
LOC_Os02g43010 VPE3, REP-2
LOC_Os05g51570 VPE4
LOC_Os01g02000 PHO1;1
LOC_Os02g56510 PHO1;2
LOC_Os05g48390 PHO2, LTN1, RLS1
LOC_Os06g40120 SPX1
LOC_Os02g10780 SPX2
LOC_Os10g25310 SPX3
LOC_Os03g61200 SPX4
LOC_Os03g29250 SPX5
LOC_Os03g05334 IPS1
LOC_Os09g32840 PAP2
LOC_Os01g56880 PAP10a
LOC_Os12g44020 PAP10c, PAP27b, NPP1
LOC_Os11g05400 PAP21b
LOC_Os06g43640 PAP26
LOC_Os02g22020 NIGT1
LOC_Os01g04920 SQD2.2
LOC_Os02g55910 MGD2
LOC_Os08g20420 MGD3
LOC_Os03g40670 GDPD5
AWC
AWN
AWM
EWC
EWM
APN
APM
AWN/AWC
AWM/AWC
EWC/AWC
EWM/EWC
APM/APN

Figure S9. Heatmap summaries of PHT and phosphate-related genes across conditions.

(A) shows Z-score–scaled variance-stabilized (VST) expression values based on group means for each condition (AWC, AWN, AWM, EWC, EWM, APN, APM). Z-scores were calculated per gene across conditions, where yellow indicates higher-than-average expression and blue indicates lower-than-average expression. **(B)** displays DESeq2 log2 fold changes (Log2FC) for five contrasts (AWN/AWC, AWM/AWC, EWC/AWC, EWM/EWC, APM/APN). Color intensity reflects the magnitude and direction of Log2FC (yellow = up-regulation, blue = down-regulation). Asterisks denote statistical significance: **red** stars indicate adjusted p-value (padj) < 0.05, while **grey** stars indicate nominal p-value < 0.05 but padj ≥ 0.05. Gene identifiers and functional annotations are shown on the right. Grey cells indicate missing values (NA), resulting from insufficient expression or lack of variance for a given gene in the corresponding condition or contrast.

Table S1: Primer sequences used for real-time RT-PCR gene expression analysis, from Yang et al. (2012) Plant Cell. Forward and reverse primer sequences are shown in the 5'→3' direction. Cyclophilin2 and Ubiquitin were used as reference genes.

Gene	Forward primer (5'→3')	Reverse primer (5'→3')
PT2	GACGAGACCGCCCAAGAAG	TTTTCAGTCACTCACGTCGAGAC
PT6	CCGCCCCTGCAAACTGTA	CAACTGGCGGTTTCTTCGAT
PT11	CATATCCCAGATGAGCGTATCATG	GAGAAGTTCCCTGCTTCAAGCA
PT13	CTTCTCCATCTCCCTTGTCG	TTTTGTCGCCTAGCCAGCC
Cyclophilin2	GTGGTGTTAGTCTTTTTATGAGTTCGT	ACCAAACCATGGGCGATCT
Ubiquitin	CATGGAGCTGCTGCTGTTCTAG	CAGACAACCATAGCTCCATTGG

SI Text

Code Availability – R Script for Principal Component Analysis

The R script below was used to generate the principal component analysis (PCA) plot shown in Fig.3.

```
# ==============================================================================
# RNA-seq Differential Expression and PCA Analysis Pipeline
# ==============================================================================

# 1. LOAD REQUIRED LIBRARIES
# ------------------------------------------------------------------------------
# Core Bioconductor packages
library(DESeq2)
library(sparseMatrixStats)
library(SparseArray)
library(DelayedMatrixStats)

# Tidyverse and visualization packages
library(tidyverse)
library(ggplot2)
library(ggforce)
library(cowplot)
library(DEGreport)

# 2. DATA IMPORT AND DESEQ2 OBJECT CREATION
# ------------------------------------------------------------------------------
# Read raw gene count matrix
gene_count_table <- read.csv("CO2_RNAseq_countmatrix.csv")
message("Initial count matrix dimensions: ", paste(dim(gene_count_table), collapse = " x
"))

# Extract annotation data (first 6 columns)
rice_annotation <- as_tibble(gene_count_table[, 1:6])

# Format count matrix (setting rownames and extracting sample columns 7 to 27)
gene_count_matrix <- column_to_rownames(gene_count_table[, c(1, 7:27)], var = "id")

# Create DESeqDataSet (Ensure 'sampleTable' and 'condition_colours' are predefined in
your environment)
# Example initialization if not run previously:
# sampleTable <- read.csv("sample_metadata.csv")
# condition_colours <- c("awc" = "#7F7F7F", "treatment1" = "#1F77B4", ...)

dds <- DESeqDataSetFromMatrix(countData = gene_count_matrix,
                              colData = sampleTable,
                              design = ~ condition)

# 3. INITIAL QUALITY CONTROL (PRE-FILTERING)
# ------------------------------------------------------------------------------
dir.create("plots/DESEq2_QC", recursive = TRUE, showWarnings = FALSE)

# Custom placeholder metrics/plots from your workflow
# Note: Ensure functions like plot_total_counts, yplot_library_complexity,
# and plot_gene_detection are loaded from your local package utils if custom.
try({
  plot_total_counts(dds)
  ggsave(filename = "plots/DESEq2_QC/total_counts.pdf", width = 5, height = 5)

  yplot_library_complexity(dds)
  ggsave(filename = "plots/DESEq2_QC/library_complexity.pdf", width = 5, height = 5)

  plot_gene_detection(dds)
```

```
  ggsave(filename = "plots/DESEq2_QC/gene_detection.pdf", width = 5, height = 5)
}, silent = TRUE)

# Plot Raw Count Distribution
counts_raw <- counts(dds)
counts_df <- as.data.frame(counts_raw)
counts_long <- counts_df %>%
  pivot_longer(cols = everything(), names_to = "name", values_to = "count") %>%
  merge(sampleTable, by = "name")

ggplot(counts_long, aes(x = name, y = count, fill = condition)) +
  geom_violin(alpha = 0.6, linewidth = 0.25) +
  geom_boxplot(width = 0.2, fill = "white", color = "#4D4D4D", outlier.shape = NA,
linewidth = 0.2) +
  labs(x = "Sample", y = "Counts", title = "DESeq2 Raw Count Distribution") +
  scale_fill_manual(name = "Condition", values = condition_colours) +
  geom_vline(xintercept = c(0), linetype = "dashed") +
  scale_x_discrete(breaks = sampleTable$name, labels = sampleTable$name, limits =
sampleTable$name, name = "Sample") +
  scale_y_log10(labels = scales::comma_format(scale = 10)) +
  theme_minimal()
ggsave(filename = "plots/DESEq2_QC/DESEq2_counts_boxplot.pdf", width = 7, height = 4)

# 4. PRE-FILTERING LOW-ABUNDANCE GENES
# ----------------------------------------------------------------------------
# Filter out low-expressed genes based on experimental parameters
# Optional custom package function call if available: filter_genes(dds, min_count = 5,
min_rep = 3)
keep <- rowSums(counts(dds)) >= 10
dds <- dds[keep, ]
message("Dimensions after filtering (rowSums >= 10): ", nrow(counts(dds)), " genes.")

# Factor the DESeq DataSet and specify reference baseline
dds$condition <- factor(dds$condition, levels = levels(sampleTable$condition))
dds$condition <- relevel(dds$condition, ref = "awc")

# 5. DIFFERENTIAL EXPRESSION ANALYSIS & NORMALIZATION
# ----------------------------------------------------------------------------
dds <- DESeq(dds, parallel = TRUE)

# Export counts matrices
dir.create("gene_count_tables", showWarnings = FALSE)
write.csv(counts(dds, normalized = FALSE), file = "gene_count_tables/DESeq2_counts.csv")

normalized_counts <- counts(dds, normalized = TRUE)
write.csv(normalized_counts, file = "gene_count_tables/DESeq2_normalized_counts.csv")

# Plot Normalised Count Distribution
counts_df_norm <- as.data.frame(normalized_counts)
counts_long_norm <- counts_df_norm %>%
  pivot_longer(cols = everything(), names_to = "name", values_to = "count") %>%
  merge(sampleTable, by = "name")

ggplot(counts_long_norm, aes(x = name, y = count, fill = condition)) +
  geom_violin(alpha = 0.6, linewidth = 0.25) +
  geom_boxplot(width = 0.2, fill = "white", color = "#4D4D4D", outlier.shape = NA,
linewidth = 0.2) +
  labs(x = "Sample", y = "Counts", title = "DESeq2 Normalised Count Distribution") +
  scale_fill_manual(name = "Condition", values = condition_colours) +
  geom_vline(xintercept = c(0), linetype = "dashed") +
  scale_x_discrete(breaks = sampleTable$name, labels = sampleTable$name, limits =
sampleTable$name, name = "Sample") +
  scale_y_log10(labels = scales::comma_format(scale = 10)) +
  theme_minimal()
ggsave(filename = "plots/DESEq2_QC/DESEq2_normalised_counts_boxplot.pdf", width = 7,
height = 4)
```

```
# 6. VARIANCE STABILIZING TRANSFORMATION (VST)
# -------------------------------------------------------------------------------
vsd <- vst(dds, blind = FALSE)
vsd_counts <- assay(vsd)
write.csv(vsd_counts, file = "gene_count_tables/DESeq2_VSD_counts.csv")

# Plot VSD Normalised Distribution
vsd_counts_df <- as.data.frame(vsd_counts)
vsd_long <- vsd_counts_df %>%
  pivot_longer(cols = everything(), names_to = "name", values_to = "count") %>%
  merge(sampleTable, by = "name")

ggplot(vsd_long, aes(x = name, y = count, fill = condition)) +
  geom_violin(alpha = 0.6, linewidth = 0.25) +
  geom_boxplot(width = 0.2, fill = "white", color = "#4D4D4D", outlier.shape = NA,
linewidth = 0.2) +
  labs(x = "Sample", y = "Counts", title = "VSD Normalised Count Distribution") +
  scale_fill_manual(name = "Condition", values = condition_colours) +
  geom_vline(xintercept = c(0), linetype = "dashed") +
  scale_x_discrete(breaks = sampleTable$name, labels = sampleTable$name, limits =
sampleTable$name, name = "Sample") +
  scale_y_log10(labels = scales::comma_format(scale = 10)) +
  theme_minimal()
ggsave(filename = "plots/DESEq2_QC/VSD_normalised_counts_boxplot.pdf", width = 7, height
= 4)

# 7. HIGH-DIMENSIONAL PRINCIPAL COMPONENT ANALYSIS (MANUAL OVERRIDE)
# -------------------------------------------------------------------------------
dir.create("plots/PCA", recursive = TRUE, showWarnings = FALSE)

vsd_matrix <- as.matrix(vsd_counts)
ntop <- 2000
rv <- rowVars(vsd_matrix)
select <- order(rv, decreasing = TRUE)[seq_len(min(ntop, length(rv)))]
mat <- t(vsd_counts[select, ])
pca <- prcomp(mat)
summary_pca <- summary(pca)
pca_loadings <- unlist(as.vector(as.data.frame(summary_pca$importance)[2, ]))

scores <- as.data.frame(pca$x)
scores <- rownames_to_column(scores, var = "name") %>% as_tibble()
scores <- left_join(scores, sampleTable, by = "name")

# Publication Ready Plot: PC1 vs PC2
ggplot(scores, aes(PC1, PC2, color = condition, fill = condition, shape = inoculation)) +
  xlab(paste0("PC1 (", round(pca_loadings[1] * 100, 1), "% variance)")) +
  ylab(paste0("PC2 (", round(pca_loadings[2] * 100, 1), "% variance)")) +
  scale_x_continuous(limits = c(-60, 50), expand = c(0, 0)) +
  scale_y_continuous(limits = c(-40, 50), expand = c(0, 0)) +
  ggforce::geom_mark_ellipse(aes(color = condition, fill = condition), alpha = 0.2, size
= 0.5) +
  geom_point(alpha = 0.8, size = 3) +
  geom_vline(xintercept = 0, linetype = "dashed", color = "gray50", size = 0.7) +
  geom_hline(yintercept = 0, linetype = "dashed", color = "gray50", size = 0.7) +
  theme_minimal(base_size = 14) +
  theme(
    axis.title = element_text(size = 14, face = "bold"),
    axis.text = element_text(size = 12),
    legend.title = element_text(size = 12, face = "bold"),
    legend.text = element_text(size = 11),
    panel.grid = element_blank(),
    panel.border = element_rect(color = "black", fill = NA, size = 1)
  ) +
  scale_color_manual(name = "Condition", values = condition_colours) +
  scale_fill_manual(name = "Condition", values = condition_colours) +
  scale_shape_manual(name = "Inoculation", values = c(21, 22, 23, 24))
```

```
ggsave(filename = "plots/PCA/PCA_PC1_PC2.pdf", width = 7, height = 5)

# Publication Ready Plot: PC1 vs PC3
ggplot(scores, aes(PC1, PC3, color = condition, fill = condition, shape = inoculation)) +
  xlab(paste0("PC1: ", round(pca_loadings[1] * 100, 1), "% variance")) +
  ylab(paste0("PC3: ", round(pca_loadings[3] * 100, 1), "% variance")) +
  scale_x_continuous(limits = c(-60, 50)) +
  scale_y_continuous(limits = c(-40, 50)) +
  ggforce::geom_mark_ellipse(aes(color = condition, fill = condition), alpha = 0.2) +
  geom_point(alpha = 1, size = 2) +
  geom_vline(xintercept = c(0), linetype = "dashed") +
  geom_hline(yintercept = c(0), linetype = "dashed") +
  scale_color_manual(name = "Condition", values = condition_colours) +
  scale_fill_manual(name = "Condition", values = condition_colours) +
  theme_minimal()
ggsave(filename = "plots/PCA/PCA_PC1_PC3.pdf", width = 6, height = 4)

# 8. ADDITIONAL ADVANCED DIAGNOSTICS (DEGREPORT & CLUSTERING)
# ------------------------------------------------------------------------------
# Sample correlation heatmap
set.seed(1)
try({
  pdf(file = "plots/DESEq2_QC/correlation_map.pdf", width = 10, height = 8)
  plot_sample_clustering(vsd, anno_vars = c("genotype", "CO2", "inoculation"), distance =
"euclidean")
  dev.off()
}, silent = TRUE)

# Replicate MA multi-panel variability checks
try({
  ma_plots <- plot_sample_MAs(vsd, group = "condition")
  cowplot::plot_grid(plotlist = ma_plots, ncol = 3)
  ggsave(filename = "plots/DESEq2_QC/replicate_variability.pdf", width = 20, height = 40)
}, silent = TRUE)

# Statistical validation diagnostics via DEGreport
try({
  counts_df_deg <- counts(dds, normalized = TRUE)
  res_deg <- results(dds)
  design_deg <- as.data.frame(colData(dds))

  degCheckFactors(counts_df_deg)
  ggsave(filename = "plots/DESEq2_QC/degCheckFactors.pdf", width = 5, height = 5)

  degQC(counts_df_deg, design_deg[["condition"]], pvalue = res_deg[["pvalue"]])
  ggsave(filename = "plots/DESEq2_QC/degQC.pdf", width = 10, height = 10)

  resCov <- degCovariates(log2(counts(dds) + 0.5), colData(dds))
  ggsave(filename = "plots/DESEq2_QC/degCovariates.pdf", width = 10, height = 4)

  pdf(file = "plots/DESEq2_QC/degCorCov.pdf", width = 5, height = 5)
  degCorCov(colData(dds))
  dev.off()
}, silent = TRUE)

message("Pipeline completed successfully! Figures saved to 'plots/' and tables saved to
'gene_count_tables/'.")
```

Supplemental Data Legends

Dataset S1. Normalized RNA-seq read counts across experimental conditions.

Dataset S2: Co-expression modules

Dataset S2A: Co-expression modules associated with Fig. 4. Partitioning of genes into 10 discrete co-expression modules (Module_1 to Module_10) identified, along with their respective MSU IDs, functional annotations, and established gene nomenclature, where applicable.

Dataset S2B: Differential expression categories across mycorrhizal and environmental treatments associated with Fig.5. Categorisation of rice genes based on transcriptomic responsiveness to symbiosis under ambient and/or elevated CO_2 conditions

Dataset S2C. Co-expression module partitioning AM-regulated genes under ambient and elevated CO_2 conditions, associated with Fig. 6a–c.

Dataset S2D. Assessment of PT11-dependency in symbiotic gene expression associated with Fig. 6d–f. Columns outline specific gene IDs, manual/final functional annotations, average expression values in mycorrhizal roots (APM_mean), and the final classification into "PT11-Dependent" or "PT11-Independent" gene expression profile.

Dataset S3: Cross-reference gene lists

Dataset S3A: PHR2-regulated gene candidates. List of PHR2 target genes derived from Shi et al., 2022 (*New Phytologist*). Includes $\log_2$ fold changes, raw *P* values, Benjamini-Hochberg adjusted false discovery rates (*Q* values), and the direction of PHR2-mediated transcriptional regulation ("up" or "down").

Dataset S3B. SMAX1-regulated gene candidates. Rice target genes modulated by Suppressor of MAX2 1 (SMAX1) sorted into operational categories (I–IV) as established by Choi et al., 2020 (*Nature Communications*).

Dataset S4A.

Dataset S4A. Pairwise DEG analysis of non-mycorrhizal split-root half vs. non-colonized control plants, wild-type, under ambient CO_2 (awn_vs_awc). Delineates systemic signalling and transcriptomic changes induced by arbuscular mycorrhizal colonisation under ambient CO_2.

Dataset S4B. Pairwise DEG analysis of ambient conditions in the mycorrhizal split-root half vs. completely non-colonized control plants, wild-type, under ambient CO_2 (awm_vs_awc). Captures the comprehensive root transcriptomic response to arbuscular mycorrhizal symbiosis under standard ambient CO_2 relative to completely uncolonized, naïve plants.

Dataset S4C. Pairwise DEG analysis of ambient conditions in the mycorrhizal split-root half vs. ambient non-mycorrhizal split-root half in wild-type (awm_vs_awn). Isolates local, direct mycorrhiza-induced transcription alterations by directly subtracting systemic/background changes occurring across the non-colonised hemisphere of the split-root system under under ambient CO_2.

Dataset S4D. Pairwise DEG analysis of elevated vs. ambient CO_2 conditions in completely non-colonised wild-type plants (ewc_vs_awc). Identifies genes exclusively responsive to atmospheric CO_2.

Dataset S4E. Pairwise DEG analysis of elevated CO_2 conditions in the mycorrhizal split-root half completely non-colonised wild-type plants (ewm_vs_ewc). Captures transcriptomic reprogramming induced by direct arbuscular mycorrhizal colonization under an elevated atmospheric CO_2 regime.

Dataset S4F. Pairwise DEG analysis of elevated vs. ambient CO_2 conditions on mycorrhizal split-root half in wild-type (ewm_vs_awm). Captures effect of elevated CO_2 on the mycorrhizal root transcriptome.

Dataset S4G. Pairwise DEG analysis of *pt11-1* versus wild-type roots from non-mycorrhizal split-root half under ambient CO_2 conditions (apn_vs_awn). Identifies the misregulated transcriptome disrupted by the *pt11-1* loss-of-function mutation.

Dataset S4H. Pairwise DEG analysis in the mycorrhizal vs non-mycorrhizal split-root half in the *pt11-1* mutant, under ambient CO_2 conditions (apm_vs_apn). Highlights local, direct expression dynamics driven by fungal inoculation within the *pt11-1* mutant context, where normal functional arbuscule development is impaired.

Dataset S4I. Pairwise DEG analysis of wild-type vs. *pt11* mutant in the mycorrhizal split-root half (awm_vs_apm). Reveals the mycorrhiza regulated genes that occur in wild-type but not in *pt11-1* mutant.
